# Limited locomotive ability relaxed selective constraints on molluscs mitochondrial genomes

**DOI:** 10.1038/s41598-017-11117-z

**Published:** 2017-09-06

**Authors:** Shao’e Sun, Qi Li, Lingfeng Kong, Hong Yu

**Affiliations:** 1 Key Laboratory of Mariculture, Ministry of Education, Ocean University of China, Qingdao, 266003 China; 2 Laboratory for Marine Fisheries Science and Food Production Processes, Qingdao National Laboratory for Marine Science and Technology, Qingdao, China

## Abstract

Mollusca are the second largest phylum in the animal kingdom with different types of locomotion. Some molluscs are poor-migrating, while others are free-moving or fast-swimming. Most of the energy required for locomotion is provided by mitochondria via oxidative phosphorylation. Here, we conduct a comparative genomic analysis of 256 molluscs complete mitochondrial genomes and evaluate the role of energetic functional constraints on the protein-coding genes, providing a new insight into mitochondrial DNA (mtDNA) evolution. The weakly locomotive molluscs, compared to strongly locomotive molluscs, show significantly higher Ka/Ks ratio, which suggest they accumulated more nonsynonymous mutations in mtDNA and have experienced more relaxed evolutionary constraints. Eleven protein-coding genes (*CoxI*, *CoxII*, *ATP6*, *Cytb*, *ND1-6*, *ND4L*) show significant difference for Ka/Ks ratios between the strongly and weakly locomotive groups. The relaxation of selective constraints on *Atp8* arise in the common ancestor of bivalves, and the further relaxation occurred in marine bivalves lineage. Our study thus demonstrates that selective constraints relevant to locomotive ability play an essential role in evolution of molluscs mtDNA.

## Introduction

In all eukaryotic taxa, mitochondria play a vital role as the power sources of cells by providing up to 95% cell energy through oxidative phosphorylation (OXPHOS)^1^. Mitochondrial OXPHOS has two primary physiological functions: adenosine triphosphate (ATP) production, that is used for locomotion and other energy-consuming processes, and heat generation, which is mainly for endotherms^2–4^. Each mitochondrion contains its own genomic system, generally a small closed circular DNA molecule^5, 6^. Although mitochondria themselves perform different tasks, the mitochondrial genome encodes proteins associated with only one pivotal task^7^. Mitochondrial DNA (mtDNA) usually encodes 13 subunits of the protein complexes, all playing vital roles in the electron transport and ATP synthesis^5, 6^. Therefore, mitochondrial genes are extremely sensitive to energy-related selective pressures.

Over the years, hypotheses have been put forward to examine how organismal differences in key traits linked to mitochondrial biology (e.g. aerobic metabolic rate) are reflected in different selective regimes acting on the mitochondrial genome^7^. Recent studies revealed that the mtDNA of weakly locomotive bird and mammalian lineages have undergone relaxation of evolutionary constraints following the degeneration of locomotive ability^8, 9^. The energy budget influences the evolution of the mitochondrial proteins in fishes in a predictable way: increased energy demand is accompanied by evidence for stronger purifying selection^10^. Shen *et al*. identified that the mitochondrial encoded OXPHOS genes were targets of natural selection and allowed for adaptation to the huge change in energy demand that was required during the origin of flight in bats^11^. These studies provide important insight toward exploring how the metabolic requirement would impact selective constraints acting on the mitochondrial genome in vertebrates lineages; however, the full range of metabolic demands that characterize invertebrates lineages remains largely unexplored.

Mollusca are the second largest phylum of animals, with approximately 200,000 living species. As one of the major metazoan phyla, molluscs are successful in both terrestrial and aquatic (marine and freshwater) environments and have penetrated perhaps a wider range of habitats than virtually any other animal groups^12, 13^. Gastropoda is the largest molluscan class, comprising over 80% of all living molluscs. They are present in every marine environment and have successfully invaded a vast array of freshwater habitats. What’s more, gastropods are the only molluscan group that have invaded the land. They have generally retained a single appendage, the foot for crawling^13–15^. Bivalves are the second largest group of molluscs, outnumbered only by gastropods, representing one of the most common animal groups in both marine and freshwater ecosystems^16^. Bivalves attach to the substratum by three means: permanently-fixed by one valve, byssally adhered and burrowed in infaunal sediment^13, 17^. Thus, bivalves, except scallop species, have no or little motility as adults. Cephalopoda is the third most speciose class of molluscs, with all members marine^18^. Differing greatly from other molluscs, cephalopods are relatively more active and fast-swimming, with elaborate neural centers and complex behavioral patterns that allow them to be competitive and efficient predators^12, 13^. The different types of locomotion in molluscs are well adapted to their “lifestyle”, which has a crucial role in assisting them to perching, feeding, breeding and predator avoidance. Locomotion is energy-consuming, and strongly locomotive organisms, such as the flying birds, high locomotive speed mammals, (Ln *S*, loge-transformed speed *S* > 1.79), and the migratory fishes, usually require a more active metabolism than weakly locomotive organisms, such as the running and swimming birds, low locomotive speed mammals, (Ln *S*, loge-transformed speed *S* > 1.79), and the nonmigratory fishes^8, 10, 19^. According to this view, the strongly locomotive molluscs may need to maintain highly efficient energy metabolism to support their rapid movement. The mitochondrial DNA mutations are extremely sensitive to energy-related selective pressures^8, 20^. Thus, the mitochondrial DNA of the strongly locomotive molluscs would have experienced stronger selective constraints. In contrast, the weakly locomotive molluscs might have undergone relaxation of selective constraints.

The genetic consequences of the change in lifestyle associated with different locomotive abilities lead to two questions. First, if the substitution rate are varied in the mtDNA of strongly and weakly locomotive molluscs? Second, do all the mtDNA-encoding genes experience the same selection pressure? To address these questions, we conduct a comparative genomic analysis of 256 molluscs complete mitochondrial genomes and test the roles of the evolutionary constraints acted on mitochondria to provided a more complete view of mtDNA evolution.

## Results

### Ratio of nonsynonymous/synonymous nucleotide substitutions

**Figure 1:**
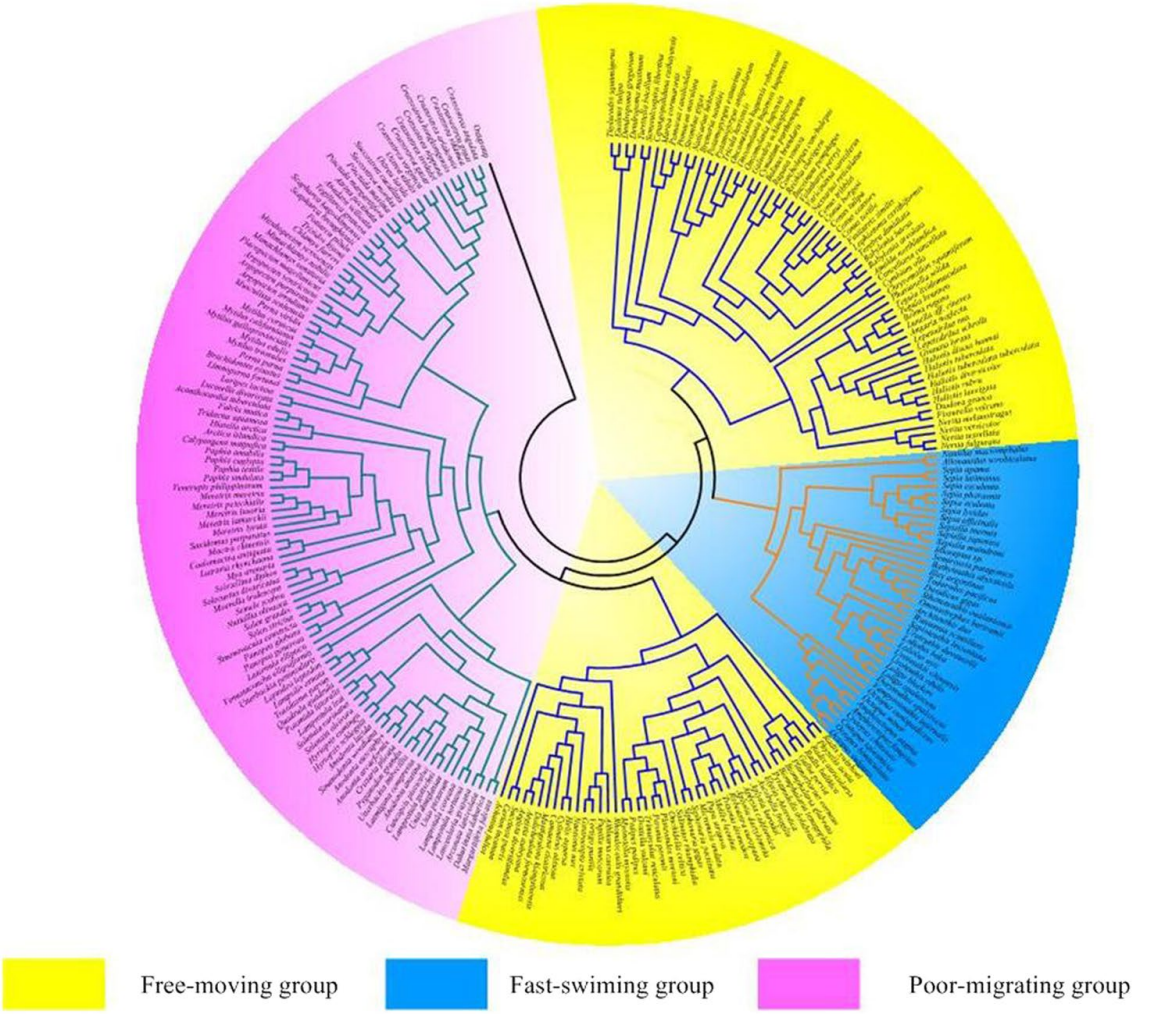
Circular Maximum-likelihood (ML) tree of the 256 molluscs used for evolutionary analysis of mitochondrial genomes. The phylogenetic tree was constructed using all 13 proten-coding genes from mitochondrial genomes. The “poor-migrating”, “free-moving” and “fast-swimming” groups are marked in yellow, blue and pink, respectively.

We assembled a data set based on the complete mitochondrial genomes of 256 molluscs (Supplementary Table 1) and constructed the maximum likelihood phylogenetic tree (Fig. 1 and Additional file 2: Fig. S1). As shown in Fig. 1, molluscs with the same locomotive styles didn not cluster together. Bivalves (marked in turquoise), such as oysters, mussels and clams, either have no/little or less motility in adults, which are cemented or byssally attached and burrowing. Gastropodas (marked in blue), such as snails, sea slugs, which retained the foot for crawling, are free-moving. Cephalopods (e.g., squids, cuttlefishes) (marked in orange), are the most active and fast-swimming. Obviously, Cephalopods move fastest among all molluscs, closely followed by Gastropodas, and Bivalves is the slowest.

**Figure 2:**
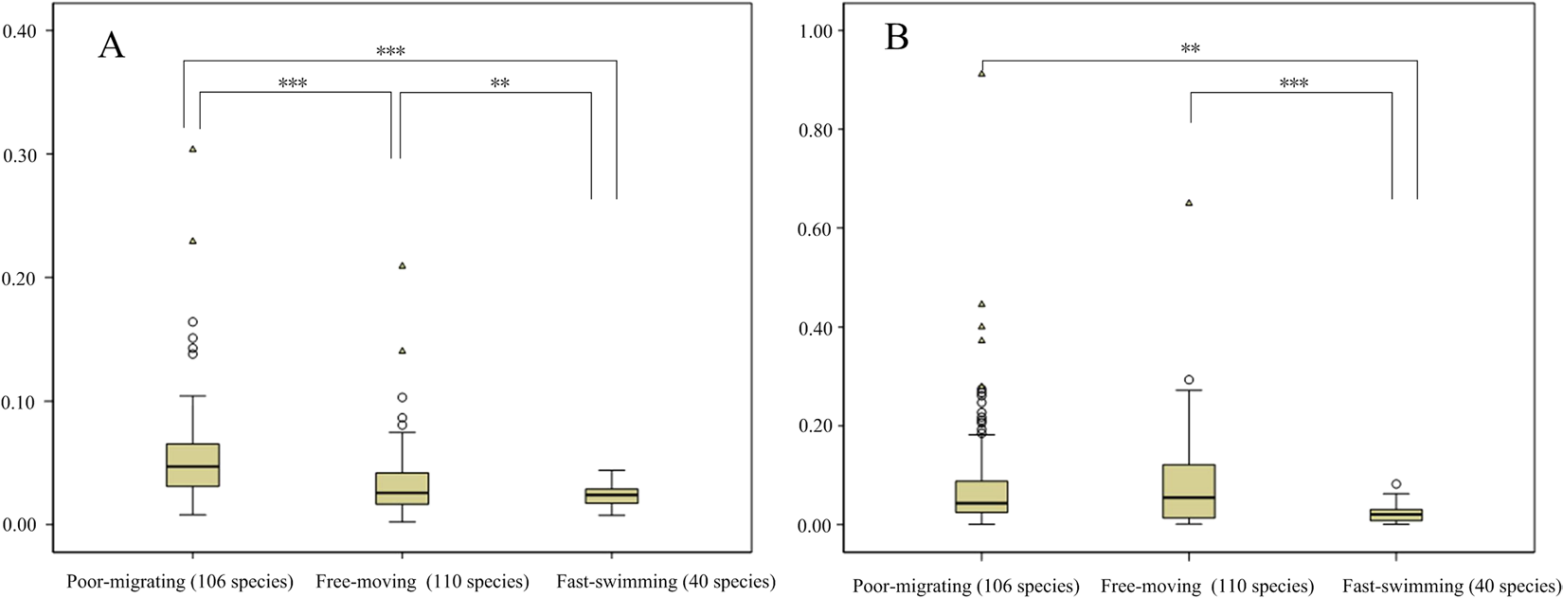
Comparisons of average Ka/Ks ratios and Ka among groups of different locomotive ability. The boxplots were constructed by IBM SPSS Statistics 19 with the outliers removed. (**A**) Mean Ka/Ks ratio comparisons among the poor-migrating, Free-moving and Fast-swimming groups; (**B**) Mean Ka value comparisons among the poor-migrating, Free-moving and Fast-swimming groups. Note: *0.01 < P < 0.05, **0.001 < P < 0.01, ***P < 0.001.

The selective constraints acting on the protein-coding sequences is commonly measured by calculating the ratio of nonsynonymous (change in amino acid)/synonymous (silent) substitutions (Ka/Ks). To determine if the mtDNA of molluscs with different locomotive ability experienced differing selective constraints, we calculated the Ka/Ks values for the mt protein-coding genes associated with terminal branches (that is, Ka/Ks between modern species and their most recent reconstructed ancestor). The mitochondrial data set of 256 molluscs (listed in Supplementary Table 1) were first divided into “poor-migrating”, “free-moving” and “fast-swimming” groups, to represent groups of different locomotive ability with different energy demands. The “poor-migrating” group had the highest mean value for both Ka/Ks and Ka (Fig. 2A and B) with the mean Ka/Ks of being 0.0559, a value significantly greater than that of the “free-moving” (0.0340) and “fast-swimming” groups (0.0240) (*P* < 0.001 and *P* < 0.001, respectively). The Ka/Ks ratios was also significantly different between “free-moving” and “fast-swimming” groups (*P* = 0.035). The mean Ka of the “poor-migrating” group (0.0858) was significantly higher than that of the “fast-swimming” group (0.0228) (*P* = 0.001), and higher than that of the “free-moving” group (0.0809), although it was not significant (*P* = 0.733). The “free-moving” group has a significantly greater Ka than the “fast-swimming” group (*P* < 0.001). These results demonstrated that the mitochondrial protein-coding genes of molluscs (“poor-migrating” or “free-moving” group) with lower energy demands accumulated more nonsynonymous mutations compared with these (“fast-swimming” group) with higher energy demands, thus have experienced relaxation of selective pressures.

Differences in synonymous mutation rate have biased the results. The average Ks in the “poor-migrating” group (2.1142) and the “free-moving” group (2.5792) are significantly larger than that of the “fast-swimming” group (0.9898; *P* < 0.001, *P* = 0.02) (Supplementary Table 1). The greater Ks in the weakly locomotive group may lead to smaller Ka/Ks ratios, making our results more conservative. Hence, these analyses suggested that the larger Ka/Ks values in the weakly locomotive molluscs were not simply result from the differences in synonymous mutation rates (Ks).

The analyses above suggest that the Ka/Ks variation among groups depend upon the energy demands for locomotion. To determine whether these trends result from the energetic functional constraints, or they reflect a general mode of molecular evolution for these species, we analyzed one nuclear gene (histone H3) from 144 molluscs that is not involved in energy metabolism (available in Supplementary Table 2.), using the same criteria as above. The histone H3 gene would be equally affected by any general molecular evolutionary pressure like the mitochondrial-encoded genes, but they should not be subjected to constraints due to energy demand. The Ka/Ks ratio of the “poor-migrating” group was not significantly higher than that of neither the “free-moving” nor “fast-swimming” groups (0.0777 vs. 0.0204, *P* = 0.102; 0.0777 vs. 0.0162, *P* = 0.071, respectively) (Supplementary Table 2). Similarly, the mean Ka of “poor-migrating” group was also not greater than that of neither “free-moving” nor “fast-swimming” groups (0.0006 vs. 0.0004, *P* = 0.456; 0.0006 vs. 0.0014, *P* = 0.162, respectively). This bias may be due to the difference in the number of species in the mtDNA and nuclear gene data sets. To avoid this bias, we repeated the above analysis using 16 overlapping species (species for which both mtDNA and nuclear genes available), including six poor-migrating species, five free-moving species and five fast-swimming species. For the mtDNA genes data set, we find a greater Ka/Ks ratio for the “poor-migrating” group compared with either the “free-moving” or “fast-swimming” groups (0.1342 vs. 0.0413, P = 0.050; 0.1342 vs. 0.0183, P = 0.047, respectively). The Ka/Ks ratios between the “free-moving” and “fast-swimming” groups were also significantly different (*P* < 0.001), whereas the ratio for histone H3 gene was not significantly different among the three groups *(P* = 0.447, *P* = 0.474, and *P* = 0.407). Besides that, the mean Ka of the mtDNA genes in “poor-migrating” group was significantly larger than that in the “free-moving” and “fast-swimming” groups (0.0658 vs. 0.0346, *P* = 0.05; 0.0658 vs. 0.0214, *P* = 0.020, respectively), whereas this pattern was not observed for the nuclear gene data set. Thus, mtDNA genes tend to have accumulated more mutations and show increased Ka/Ks ratios. These results suggested that the energetic functional constraints likely influence the evolution of mitochondrial-encoded proteins in molluscs.

### Relaxation of selective constraint on *Atp8* gene

**Figure 3:**
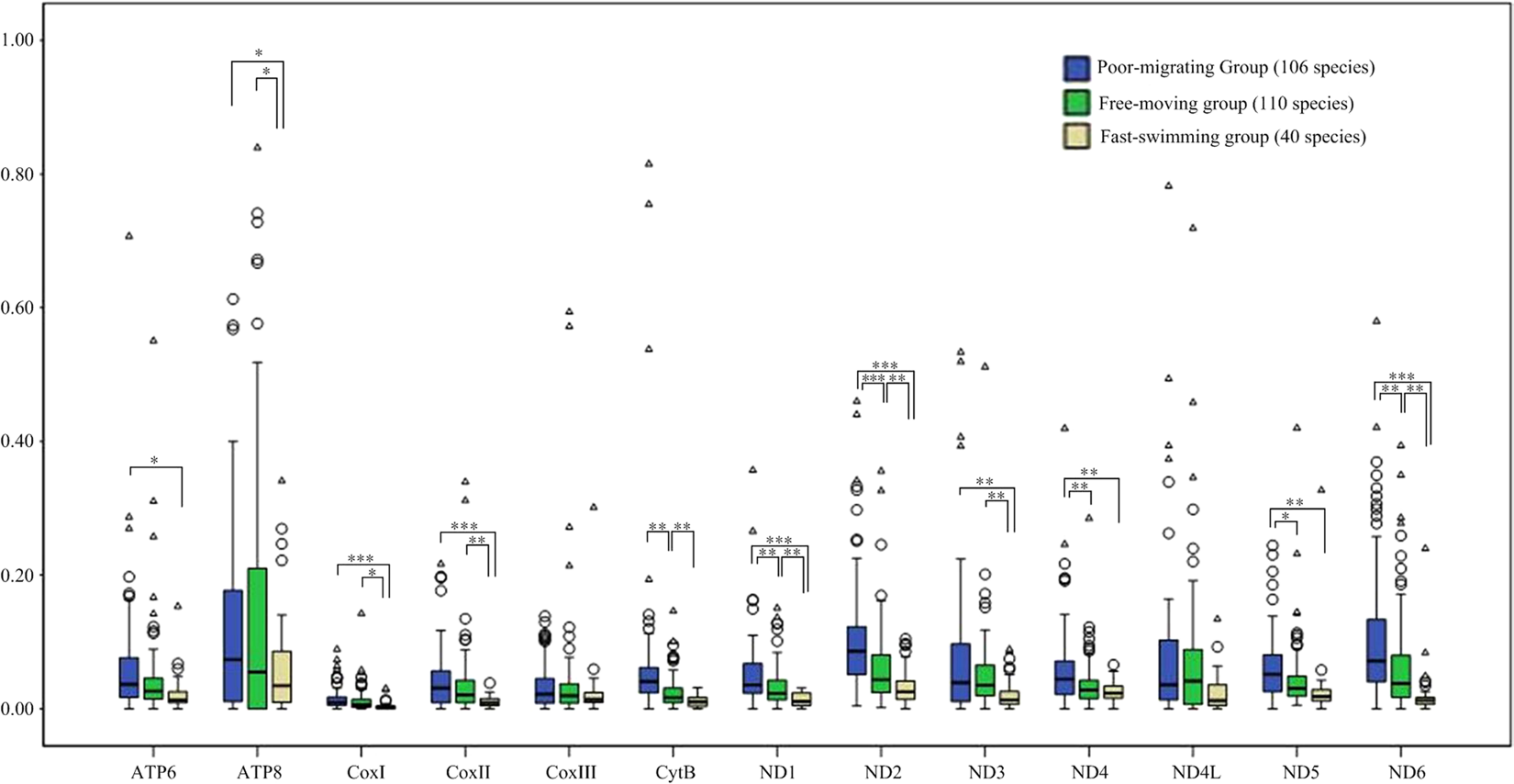
Comparisons of average Ka/Ks ratios of the 13 protein-coding genes among the poor-migrating, Free-moving and Fast-swimming groups. Note: *0.01 < P < 0.05, **0.001 < P < 0.01, ***P < 0.001.

To identify which of the mitochondrial proteins were most influenced by the constraints of motility effects, we repeated the above analysis for each of the 13 individual genes (Supplemental Table 1). The Ka/Ks ratios varied among the different genes, demonstrating that different genes have differing nonsynonymous substitution rates depending upon energy demands (Fig. 3). All genes except *CoxIII* and *ND4L*were shown to have significantly smaller mean Ka/Ks in strongly locomotive groups. This result suggested that some genes (e.g., *CoxI*, *CoxII*, *ATP6*, *Cytb*, *ND1-6*) may have more important roles in energy production, and thus experienced stronger functional constraints.

Absence of *Atp8* gene has been suggested for most bivalves studied so far, especially marine species. We examined the *Atp8* gene for Gastropodas, Cephalopods, freshwater bivalve species and sixteen marine bivalve species, in which the *Atp8* gene was annotated (Supplementary Table 3). The average Ka/Ks ratio of *Atp8* gene in marine bivalve species is higher than that of other groups, although it was not significant (Supplementary Table 3). We thus hypothesize that the *Atp8* genes might be relaxed from selective constraints because of the change in locomotive ability and the reduced energy requirements that emerged in marine bivalve evolution.

**Figure 4:**
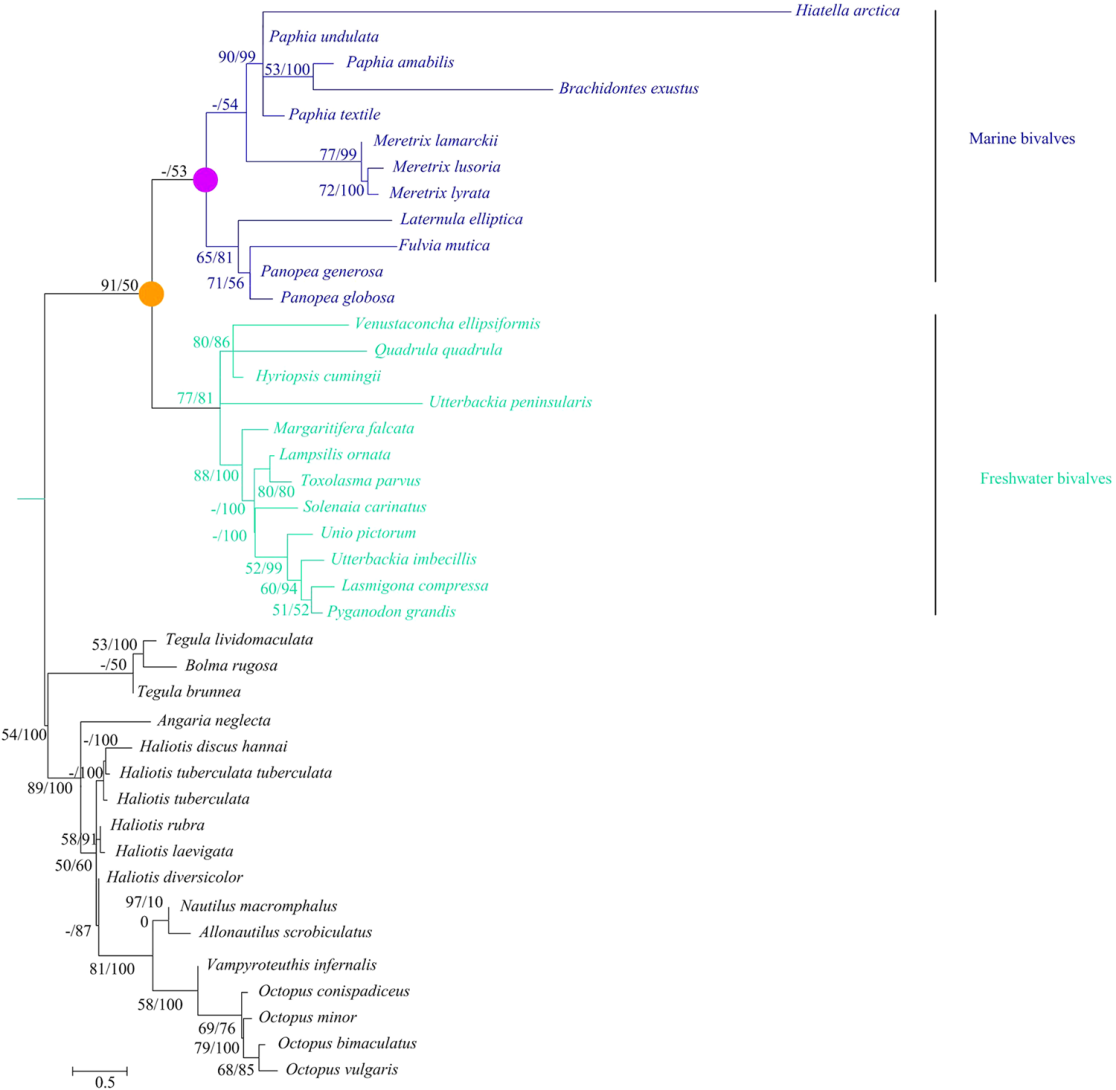
The phylogenetic tree of *Atp8* gene in molluscs under GTR + I + G substitution model of sequence evolution. Branch lengths are drawn to scale. ML bootstrap values/Bayesian posterior probabilities (>50%) are shown as numbers above the branches. The scale bar means 0.5 substitutions per site. The branch and species names colours identify the marine bivalves clade (blue) and the freshwater bivalves (turquoise). The common ancestor of bivalves and marine bivalves are marked in orange and purple circle, respectively.

When the functional relaxation in marine bivalve *Atp8* gene occur, before or after the radiation of extant marine bivalves? To explore this issue, we first constructed phylogenetic analysis using *Atp8*, as well as *CoxI*, *CoxII*, *ATP6* and *Cytb* genes, and confirmed that the two groups (marine and freshwater bivalves) are well-separated from their common ancestor (Fig. 4, Supplementary Fig. 1). Then the ratios of nonsynonymous and synonymous nucleotide substitution rates (ω = Ka/Ks) of *Atp8* in bivalves were estimated using a likelihood approach. The likelihood tests were computed on three data sets. Data set I included 17 nonbivalve molluscs and one ancestral sequence of all bivalves. Data set II contained 29 nonmarine bivalve molluscs and one ancestral sequence of all marine bivalves. Data set III included 29 nonmarine bivalve molluscs and 12 marine bivalves (Table 1). First, we analyzed data set I under the supposition of a uniform ωin all branches (model A); ω was estimated to be 0.07, indicating an overall purifying selection on *Atp8* in molluscs. Supposing the ancestral branch of all bivalves (orange circle in Fig. 4) has ω2 and other branches have ω1 (model B), we estimated ω1 = 0.07, ω2 = 0.71. The model B fits the data significantly better than model A (P = 0.021), suggesting the common ancestor of all bivalves has a significantly greater ω than other molluscs. This result supports the hypothesis that a relaxation of functional constraint started from the common ancestor of all bivalves. Interestingly, this trend is well reflected by the elevated branch length of the ancestral branch leading to bivalves (Fig. 4). Second, we examined data set II and found that model C, which allowed a same ω in all branches, was significantly better than model D, which features a variation in ω between the ancestral branch of all marine bivalves and other branches (P = 0.037). This result suggests a second wave of more complete relaxation of functional constraint after the divergence of marine and freshwater bivalves. Third, analyzed data set III and allowed a variation ω between the ancestral branch of all marine bivalves (purple circle in Fig. 4) and all branches connecting 12 marine bivalves (model D). The ω of the ancestral branch of marine bivalves was not significantly lower than that of other marine bivalve branches (P = 0.01) after comparing model F with model E, the latter of which is a model assuming the same ω for all marine bivalve branches. These findings indicate similar levels of selective pressure on the *Atp8* between the ancestral branch and branches connecting the twelve examined marine bivalves.

**Table 1:** Likelihood Ratio Tests of selective pressures on *Atp8* gene in molluscs.

Models	ω (Ka/Ks)	Comparisons	p value (LRT)
Data set I: 18 *Atp8* sequences (17 nonbivalve molluscs plus the ancestral sequence of all bivalves)			
A. All branches have the same ω	ω = 0.07	B vs. A	**0.021**
B. Ancestral branch of all bivalves has ω2 and other branches have ω1	ω1 = 0.07, ω2 = 0.71		
Data set II: 30 *Atp8* sequences (29 nonmarine bivalve molluscs plus the ancestral sequence of all marine bivalves)			
C. All branches have the same ω	ω = 0.09	D vs. C	**0.037**
D. Ancestral branch of all marine bivalves has ω2 and other branches have ω1	ω1 = 0.09, ω2 = 0.24		
Data set III: 41 *Atp8* sequences (29 nonmarine bivalve molluscs plus 12 marine bivalves)			
E. Ancestral branch of all marine bivalves and branches connecting 12 marine bivalves have ω2, whereas other branches have ω1	ω1 = 0.09, ω2 = 0.20	F vs. E	0.145
F. Ancestral branch of all marine bivalves has ω3, branches connecting 12 marine bivalves have ω2, and other branches have ω1	ω1 = 0.09, ω2 = 0.24, ω3 = 0.14		

Significant *P* values (<0.05) are indicated in bold.

In addition, we found that a model assuming a variation in ω between marine and freshwater bivalves is significantly better than a simpler model estimating a same ω for both lineages of bivalves (P = 0.045, Chi-square test). This finding suggests that different levels of selective pressure acting on marine and freshwater bivalves.

## Discussion

In this study, we analyzed 256 molluscs mitochondrial genomes to determine whether the mtDNA of different groups of molluscs experienced different strength of selective pressures during the evolution through calculating the ratio of rates of nonsynonymous substitution over synonymous substitutions (Ka/Ks), a traditional measure of the strength of selection on proteins^21^. The median Ka/Ks ratio differs sharply across the three lineages, with the “free-moving” lineage falling between “poor-migrating” and “fast-swimming” lineages. It also demonstrated that the weakly locomotive species accumulate more nonsynonymous mutations.

An alternative hypothesis to explain our finding is that the functional constraints were relaxed on the mtDNA in molluscs species with lower locomotive abilities. Increases in rates of nonsynonymous substitutions generally increase ratios of radical amino acid substitutions^22^, whereas the accumulation of radical substitutions in mtDNA genes may lead to a reduction in electron-transferring respiratory chain activity^23, 24^. The mtDNA of strongly locomotive molluscs has experienced stronger purifying selection to maintain efficient energy metabolism, since they need more energy consumption during their free movement or fast swimming. In contrast, the weakly locomotive individuals are more likely to survive and reproduce with lower metabolic efficiency under similar circumstances, due to their lower requirement for energy. It has been reported that the relaxed metabolic constraints in low locomotive speed birds and mammals result the accumulation of additional nonsynonymous mutations in their mitogenome^8^. The domestication in dogs and yaks was a general relaxation of selective constraint on their mitogenome, leading to elevated level of nonsynonymous changes in mitochondrial genes than their ancestor, the gray wolf ^20, 25^. In accordance with these studies, the relaxation of energetic selective constraints may have strongly contributed to the accumulation of deleterious mutations in mitogenomes of weakly locomotive molluscs. It is also possible that the accumulation of replacement mutations in the mitochondrial genomes of weakly locomotive species may have result from positive selection. Positive selection is one of the major mechanism of adaptive evolution^11^. Weakly locomotive species would have lower dispersal ability, and many of them persist in small regions, coping with more harsh and dynamical changing environments which will influence the evolution of mitogenome. We thus deduced that positive selection may have occurred on some mitochondrial genes in weakly locomotive species to generate “good” (adapted) mitochondrial genes.

For molluscs, the locomotive ability is just one of the factors determining the natural behavior besides climate, environment and *etc*., therefore less significant differences are found in two (*CoxIII* and *ND4L*) of the thirteen individual genes from the mtDNA. In addition to energy for locomotion, mitochondria also generate heat for self-thermoregulation^10^. The heat generation efficiency in organisms is usually influenced by the climate, where individuals who live in colder zones have greater heat production to maintain or elevate their body temperatures^26, 27^. Maybe these two genes have more important roles in heat generation, though this has not been proved in present. However, the important role of the nuclear genes in locomotive ability of molluscs should not be ignored. Because in addition to the mitochondrial protein-coding genes, there are more than 70 nuclear genes encoding proteins involved in oxidative phosphorylation (OXPHOS). What’s more, genes from the nuclear and mitochondrial genomes must work in concert to generate a functional oxidative phosphorylation (OXPHOS) system^28, 29^.

ATP synthase is the last enzyme complex in the respiratory chain and it couples with the mitochondrial inner membrane electrochemical gradient to produce ATP directly^30–33^. In this study, among the 13 protein-coding gene, *Atp8* showed the highest Ka/Ks ratio in each groups and significantly different from each other, implying that it experienced more relaxed selective constraints than others. In other words, in *Atp8* gene, slightly deleterious mutations have greater probability to be fixed, thus it would be more likely to loss its function.

As mentioned, absence of the *Atp8* gene has been suggested for most bivalve species, especially marine species. We showed the relaxation of selective constraints on *Atp8* gene in the common ancestor of bivalves, and the further relaxation occurred in the marine bivalves lineage. This is not unexpected, since the bivalve molluscs have limited locomotive ability, and their lower energy demand for movement may have resulted in the relaxation of selective constraint on the *Atp8* gene. Unlike marine bivalve, the *Atp8* gene has been frequently identified from all the available michondrial genome of freshwater bivalves. This is likely to reflect different responses to changes in their living environment. It is possible that the functional significance of *Atp8* gene in freshwater bivalves, as well as other molluscs, may be unimportant in marine bivalves. Our finding that functional constraints are more relaxed for the *Atp8* gene in marine bivalves has several implications. First, the weak selective constraints can rendered the loss of *Atp8* gene after sufficient time of evolution. Marine bivalves have lost and continue to lose *Atp8* gene, which would result in a decrease of energy production. Second, functional relaxation may also allow the appearance of pseudogenes of *Atp8*, which may be helpful to discover the real reason for loss of *Atp8* in marine bivalves. Future studies of ATP synthesis, including the function of nuclear genes, that are still maintained in marine bivalves may provide a better understanding of how marine bivalves can survive in the ocean without *Atp8* gene. To test if a *Atp8* gene is indeed involved with locomotive ability, it may be more appropriate for future research to examine a difference in its expression between marine and freshwater bivalves.

## Material and Methods

### Source of data

Molluscs mitochondrial genomes downloaded from GenBank, which contains 256 species available in March 2016 (Supplementary Table 1). All of the protein-coding mitochondrial genes are extracted from each genome and translated into amino acid sequences. In addition, one nuclear gene (histone H3) for 144 molluscs species were downloaded from GenBank. Both the mitochondrial and nuclear data set of the molluscs species were divided into “poor-migrating”, “free-moving” and “fast-swimming” groups, to represent groups of different locomotive ability with different energy demands. The poor-migrating group included cemented and byssally adhered bivalves, which have no or little motility in adults, and those burrowed in infaunal sediment, which have less migration and do so non-sustained. The free-moving group included gastropods capable of sustained free crawling. The fast-swimming group consisted of the cephalopods species, which were fast swimmers. These three groups were referred to as the weakly and strongly locomotive groups relative to each other.

### Ratio of nonsynonymous/synonymous nucleotide substitutions

The *Atp8* gene was absent in most bivalves. Thus, we constructed a phylogenetic tree of all 12 protein-coding genes except the *Atp8* gene using a Maximum-Likelihood (ML) approach. The twelve-partitioned nucleotide sequences of protein coding genes were aligned with ClustalX^33^. Areas of dubious alignment were isolated using Gblocks^34, 35^ (default setting) and excluded from the analyses. The best-fit nucleotide substitution models for each data partitions were selected by jModelTest^36^. The nucleotide sequences of all 12 protein-coding genes were concatenated in the same order for each species.

CodeML implemented in the PAML package^37^ was used to analysis the nonsynonymous and synonymous substitution rates (Ka/Ks) of all individual data sets estimated for each branch of the phylogenetic tree. Model 1, which allows the overall substitution rate and the ratio of Ka/Ks changes to have branch-specific values, was conducted. In other words, only Ka/Ks values associated with terminal branches were used in the subsequent analyses, *i.e*. deleterious mutations (Ka/Ks) between modern species and their most recent reconstructed ancestors. Species with missing molecular data are discarded due to their most recent ancestors on the tree being smaller than 20, as a result they have a number of nonsynonymous substitutions, as mentioned by Zhang *et al*.^9^ and Sun *et al*.^10^.

### Evolutionary analysis of *Atp8* gene

Maximum likelihood (ML) and Bayesian phylogenetic trees were reconstructed with PhyML version 3.1^38^ and MrBayes version 3.1.2^39^, respectively. The phylogenetic analysis show the results of not only *Atp8* but also other protein coding genes (e.g. *CoxI*, *CoxII*, *Atp6* and *Cytb*). The best-fit nucleotide substitution models for each data partitions were selected by jModelTest^34^. For ML analysis, 1000 bootstraps were used to estimate the node reliability. MrBayes 3.1.2 ran 1 million generations to reach the convergence with the standard deviation (SD) of split frequencies lower than 0.01. The ancestral *Atp8* sequences of bivalves were reconstructed using the Bayesian approach implemented in the BASEML program from the PAML package^37^. All statistical analysis was performed by using IBM SPSS Statistics 19.

## Electronic supplementary material

Supplementary information
