# Supplementary material for: Limited locomotive ability relaxed selective constraints on molluscs mitochondrial genomes
Source: Sci Rep. 2017 Sep 6;7:10628. doi: 10.1038/s41598-017-11117-z (PMC5587578; doi:10.1038/s41598-017-11117-z)
Supplement: Supplementary file 1 — Supplementary information [file 41598_2017_11117_MOESM1_ESM.pdf]

**Limited locomotive ability relaxed selective constraints on molluscs mitochondrial genomes**

Shao'e Sun[1], Qi Li[1,2*], Lingfeng Kong[1], Hong Yu[1]

[1]*Key Laboratory of Mariculture, Ministry of Education, Ocean University of China, Qingdao 266003, China*

[2]*Laboratory for Marine Fisheries Science and Food Production Processes, Qingdao National Laboratory for Marine Science and Technology*

* Correspondence author at : Key Laboratory of Mariculture, Ministry of Education, Ocean University of China, Yushan Road 5, Qingdao 266003, China.

Tel and fax: +86-532-82031622

E-mail address: qili66@ouc.edu.cn (Q. Li)

.

**Supplementary Table 1.** List of 256 mt genomes for molluscs and their molecular traits.

| | | | Combined 12 mt genes | | | *Ka/Ks* | | | | | | | | | | | | |
|---|---|---|---|---|---|---|---|---|---|---|---|---|---|---|---|---|---|---|
| **Accession No.** | **Species** | **Group** | ***Ka/Ks*** | ***Ka*** | ***Ks*** | **Atp6** | **Atp8** | **CO1** | **CO2** | **CO3** | **Cytb** | **ND1** | **ND2** | **ND3** | **ND4** | **ND4L** | **ND5** | **ND6** |
| AB729113 | *Scapharca broughtonii* | 1 | 0.0907 | 0.0091 | 0.0998 | | | 0.0283 | 0.0157 | | 0.0059 | 0.0423 | 0.0834 | | | 0.1226 | 0.0560 | 0.0001 |
| AB809077 | *Fulvia mutica* | 1 | 0.0382 | 0.1699 | 4.4488 | 0.0364 | 0.6130 | 0.0192 | 0.0072 | 0.0339 | 0.0213 | 0.0555 | 0.0041 | 0.0026 | 0.0377 | 0.1013 | 0.0500 | 0.3063 |
| AF177226 | *Crassostrea gigas* | 1 | 0.2968 | 0.0193 | 0.0650 | 0.7069 | | 0.0124 | 0.0001 | 0.0218 | 0.0421 | 0.2656 | 0.1808 | 0.0527 | 0.1102 | 0.0001 | 0.2303 | 0.5797 |
| AB065375 | *Venerupis philippinarum* | 1 | 0.0357 | 0.2178 | 6.0958 | 0.0598 | | 0.0073 | 0.0228 | 0.0332 | 0.0277 | 0.0503 | 0.0608 | 0.0861 | 0.0227 | 1.1707 | 0.0070 | 0.3303 |
| AB271769 | *Mizuhopecten yessoensis* | 1 | 0.0584 | 0.0823 | 1.4088 | 0.0478 | | 0.0169 | 0.0889 | 0.0681 | 0.0616 | 0.0297 | 0.0875 | 0.0185 | 0.0307 | 0.0525 | 0.0412 | 0.0673 |
| KF534717 | *Laternula elliptica* | 1 | 0.1382 | 0.3724 | 2.6936 | 0.0850 | 0.0114 | 0.0636 | 0.1100 | 0.1095 | 0.0746 | 0.0678 | 0.1477 | 0.0507 | 0.1939 | 0.0662 | 0.0905 | 0.0579 |
| KF296320 | *Solenaia oleivora* | 1 | 0.0556 | 0.0286 | 0.5140 | 0.0426 | 0.1180 | 0.0017 | 0.0373 | 0.0269 | 0.0368 | 0.0359 | 0.1227 | 0.0841 | 0.0649 | 0.0559 | 0.0574 | 0.0617 |
| KC768038 | *Ostrea lurida* | 1 | 0.0370 | 0.0350 | 0.9460 | 0.0036 | 0.2918 | 0.0151 | 0.0037 | 0.0722 | 0.0598 | 0.0760 | 0.0383 | 0.0284 | 0.0181 | 0.0719 | 0.0264 | 0.1433 |
| KC153059 | *Atrina pectinata* | 1 | 0.0469 | 0.2745 | 5.8554 | 0.0113 | | 0.0265 | 0.0075 | 0.0259 | 0.0774 | 0.0248 | 0.3415 | 0.1030 | 0.0157 | 0.0238 | 0.1045 | 0.0919 |
| JQ970425 | *Perna viridis* | 1 | 0.0311 | 0.1932 | 6.2198 | 0.0019 | 0.0130 | 0.0135 | 0.0447 | 0.0548 | 0.0581 | 0.0167 | 0.0147 | 0.0100 | 0.0577 | 0.0012 | 0.0443 | 0.0696 |
| JN786377 | *Solen strictus* | 1 | 0.0331 | 0.0326 | 0.9841 | 0.0938 | | 0.0042 | 0.0044 | | 0.0334 | 0.0279 | 0.1669 | 0.0571 | 0.0048 | 0.0135 | 0.0138 | 0.0348 |
| EU145977 | *Meretrix petechialis* | 1 | 0.1025 | 0.0009 | 0.0088 | 0.0000 | | 0.0001 | 0.0001 | 0.0000 | 0.5377 | | 0.1904 | 0.0002 | 0.0002 | 0.0000 | 0.0000 | 0.0001 |
| KF030964 | *Anodonta anatina* | 1 | 0.0543 | 0.0279 | 0.5127 | 0.0496 | | 0.0090 | 0.0212 | 0.0185 | 0.0405 | 0.0548 | 0.0510 | 0.0301 | 0.0877 | 0.1430 | 0.0665 | 0.0957 |
| KF214684 | *Mimachlamys senatoria* | 1 | 0.0767 | 0.0647 | 0.8437 | 0.0218 | 0.3812 | 0.0131 | 0.0196 | 0.0253 | 0.0237 | 0.0115 | 0.0591 | 0.0866 | 0.0194 | 0.0130 | 0.0565 | 0.0233 |
| KC109779 | *Lamprotula tortuosa* | 1 | 0.0452 | 0.0182 | 0.4028 | 0.0307 | | 0.0018 | 0.0280 | 0.0201 | 0.0255 | 0.0318 | 0.0562 | 0.0935 | 0.0317 | 0.0813 | 0.0695 | 0.1482 |
| FJ986302 | *Cristaria plicata* | 1 | 0.0349 | 0.0231 | 0.6627 | 0.0429 | | 0.0027 | 0.0277 | 0.0206 | 0.0395 | 0.0291 | 0.0477 | 0.0468 | 0.0543 | 0.0948 | 0.0384 | 0.0655 |
| JQ423460 | *Coelomactra antiquata* | 1 | 0.0122 | 0.0609 | 5.0074 | 0.0006 | | 0.0030 | 0.0389 | 0.0067 | 0.0180 | 0.0178 | 0.0509 | 0.1096 | 0.0104 | 0.0583 | 0.0185 | 0.0123 |
| DQ088274 | *Placopecten magellanicus* | 1 | 0.0578 | 0.1564 | 2.7044 | 0.0641 | | 0.0299 | 0.0672 | 0.0266 | 0.0674 | 0.0296 | 0.0819 | 0.0355 | 0.0518 | 0.1251 | 0.1376 | 0.1027 |
| GQ527172 | *Mytilus californianus* | 1 | 0.0222 | 0.0112 | 0.5026 | 0.0305 | | 0.0059 | 0.0079 | 0.0019 | 0.0142 | 0.0326 | | 0.0001 | 0.0188 | 0.0284 | 0.0387 | |
| HQ283346 | *Sinanodonta woodiana* | 1 | 0.0566 | 0.0264 | 0.4663 | 0.0846 | | 0.0057 | 0.0250 | 0.0249 | 0.0407 | 0.0387 | 0.1681 | 0.2238 | 0.0578 | 0.0510 | 0.0970 | 0.0927 |

| | | | | | | | | | | | | | | | | | | |
|---|---|---|---|---|---|---|---|---|---|---|---|---|---|---|---|---|---|---|
| EU880278 | *Sinonovacula constricta* | 1 | 0.0385 | 0.1852 | 4.8116 | 0.0165 | | 0.0170 | 0.0056 | 0.0139 | 0.0433 | 0.0856 | 0.1151 | 0.1165 | 0.0141 | 0.0168 | 0.0556 | 0.1046 |
| EU715252 | *Chlamys farreri* | 1 | 0.0683 | 0.0969 | 1.4186 | 0.0742 | | 0.0147 | 0.1172 | 0.0189 | 0.0700 | 0.0239 | 0.1220 | 0.0367 | 0.0344 | 0.0311 | 0.0662 | 0.0404 |
| EU266073 | *Crassostrea hongkongensis* | 1 | 0.0486 | 0.0180 | 0.3705 | 0.0178 | | 0.0091 | 0.0068 | 0.0304 | 0.0444 | 0.0400 | 0.0448 | 0.0851 | 0.0466 | 0.0181 | 0.0715 | 0.0004 |
| AY905542 | *Crassostrea virginica* | 1 | 0.0348 | 0.0744 | 2.1364 | 0.0196 | | 0.0135 | 0.0078 | 0.0561 | 0.0259 | 0.0152 | 0.0304 | 0.0022 | 0.0126 | 0.1459 | 0.0345 | 0.0024 |
| JF274008 | *Ostrea edulis* | 1 | 0.0491 | 0.0507 | 1.0310 | 0.0023 | | 0.0308 | | 0.0577 | 0.0491 | 0.0113 | 0.0162 | | 0.0748 | 0.0487 | 0.0326 | 0.0689 |
| FJ841968 | *Saccostrea mordax* | 1 | 0.0323 | 0.0387 | 1.1964 | 0.0209 | | 0.0123 | | 0.0268 | 0.0266 | 0.0177 | 0.0511 | | 0.0414 | 0.0081 | 0.0244 | 0.1378 |
| FJ415225 | *Mimachlamys nobilis* | 1 | 0.0443 | 0.0442 | 0.9989 | 0.0887 | | 0.0130 | 0.0727 | 0.0306 | 0.0387 | 0.0150 | 0.0383 | 0.1051 | 0.0280 | 0.0070 | 0.0636 | 0.0366 |
| FJ841967 | *Crassostrea iredalei* | 1 | 0.0654 | 0.0541 | 0.8279 | 0.0238 | | 0.0095 | 0.0110 | 0.0733 | 0.1042 | 0.0609 | 0.2974 | 0.0612 | 0.0793 | 0.0221 | 0.0588 | 0.0890 |
| FJ841966 | *Crassostrea sikamea* | 1 | 0.0534 | 0.0159 | 0.2975 | 0.0350 | | 0.0001 | 0.0001 | 0.0216 | 0.0585 | 0.0286 | 0.0609 | 0.0551 | 0.0626 | 0.0001 | 0.1636 | 0.0691 |
| FJ841965 | *Crassostrea angulata* | 1 | 0.0770 | 0.0039 | 0.0507 | 0.0001 | | 0.0001 | 0.0001 | 0.1067 | 0.0314 | 0.0621 | 0.2028 | 0.4067 | 0.0724 | 0.2624 | 0.1025 | 0.0641 |
| FJ841964 | *Crassostrea ariakensis* | 1 | 0.0975 | 0.0494 | 0.5066 | 0.0032 | | 0.0193 | 0.0043 | 0.0666 | 0.1408 | 0.0597 | 0.2250 | 0.0679 | 0.0916 | 0.1069 | 0.1073 | 0.3181 |
| GU936625 | *Mytilus trossulus* | 1 | 0.0244 | 0.0134 | 0.5501 | 0.0167 | | 0.0038 | 0.0025 | 0.0001 | 0.0132 | 0.0145 | 0.0668 | | 0.0490 | 0.0145 | 0.0423 | 0.0528 |
| HQ641406 | *Hyriopsis schlegelii* | 1 | 0.0459 | 0.0115 | 0.2505 | 0.0198 | | 0.0035 | 0.0531 | 0.0105 | 0.0319 | 0.0351 | 0.0596 | | 0.0475 | 0.0001 | 0.0514 | 0.0674 |
| HQ703012 | *Solen grandis* | 1 | 0.0183 | 0.0263 | 1.4369 | 0.0160 | | 0.0010 | | 0.0055 | 0.0123 | 0.0188 | 0.0505 | 0.0109 | 0.0078 | 0.0057 | 0.0072 | 0.0179 |
| DQ632742 | *Hiatella arctica* | 1 | 0.0990 | 0.4451 | 4.4964 | 0.2868 | 0.0165 | 0.0464 | 0.1977 | 0.0127 | 0.1309 | 0.0811 | 0.1201 | 0.5193 | 0.1167 | | 0.0894 | 0.3495 |
| HM015198 | *Crassostrea nippona* | 1 | 0.0681 | 0.0441 | 0.6465 | 0.0233 | | 0.0174 | 0.0495 | 0.0908 | 0.0525 | 0.0325 | 0.0772 | 0.0722 | 0.0719 | 0.0523 | 0.1046 | 0.0740 |
| EU023915 | *Argopecten irradians* | 1 | 0.0413 | 0.0358 | 0.8675 | 0.0054 | | 0.0001 | 0.0917 | 0.0001 | 0.0107 | | 0.0282 | | 0.0170 | 0.0119 | 0.0473 | |
| HM467838 | *Pinctada margaritifera* | 1 | 0.0479 | 0.0669 | 1.3966 | 0.1973 | | 0.0034 | 0.0590 | 0.0078 | 0.0413 | 0.0559 | 0.0372 | 0.0565 | 0.0301 | | 0.0191 | 0.2886 |
| GQ452847 | *Pinctada maxima* | 1 | 0.0428 | 0.0597 | 1.3940 | 0.1112 | | 0.0126 | 0.0291 | 0.0052 | 0.0218 | 0.1095 | | 0.0042 | 0.0271 | 0.0292 | 0.0157 | 0.0835 |
| AY484747 | *Mytilus edulis* | 1 | 0.3035 | 0.0170 | 0.0561 | 0.0001 | | 0.0177 | 0.0002 | | 0.7547 | 0.0001 | 0.4599 | 0.0000 | 0.1913 | 0.0001 | 0.0452 | 1.0220 |
| AY497292 | *Mytilus galloprovincialis* | 1 | N*dN < 20 | 0.0016 | 0.0001 | 0.0000 | | 0.0001 | 0.0883 | 0.0001 | 0.0000 | 0.0001 | 0.2531 | 0.0001 | 0.0492 | 0.0000 | | |
| GU001954 | *Musculista senhousia* | 1 | 0.0579 | 0.2475 | 4.2721 | 0.0540 | | 0.0198 | 0.1767 | 0.0731 | 0.0359 | 0.1045 | 0.0650 | 0.0040 | 0.0943 | 0.1389 | 0.0559 | 0.0683 |
| GU269271 | *Paphia euglypta* | 1 | 0.0274 | 0.0375 | 1.3662 | 0.0138 | | 0.0085 | 0.0448 | 0.0227 | 0.0176 | 0.0327 | 0.0880 | 0.0178 | 0.0762 | 0.0001 | 0.0277 | 0.0646 |
| GU071281 | *Meretrix lamarckii* | 1 | 0.1010 | 0.0638 | 0.6314 | 0.0780 | 0.0001 | 0.0457 | 0.2167 | 0.1385 | 0.0828 | 0.0066 | 0.1492 | 0.0612 | 0.0442 | 0.1590 | 0.0790 | 0.0142 |

| | | | | | | | | | | | | | | | | | | |
|---|---|---|---|---|---|---|---|---|---|---|---|---|---|---|---|---|---|---|
| GQ463598 | *Meretrix meretrix* | 1 | 0.0378 | 0.0005 | 0.0129 | 0.2694 | | 0.0000 | 0.0000 | 0.0001 | 0.0001 | 0.0000 | 0.0940 | 0.1328 | 0.0001 | 0.0001 | 0.1120 | 0.0588 |
| KF363951 | *Arctica islandica* | 1 | 0.0248 | 0.1386 | 5.5958 | 0.0310 | | 0.0173 | 0.0093 | 0.0075 | 0.0232 | 0.0169 | 0.0535 | 0.0245 | 0.0270 | 0.0165 | 0.0367 | 0.1801 |
| JF969278 | *Paphia undulata* | 1 | 0.0276 | 0.0345 | 1.2511 | 0.0197 | 0.0001 | 0.0088 | 0.0325 | | 0.0113 | 0.0316 | 0.0492 | 0.0106 | 0.0141 | | 0.0361 | 0.0311 |
| JF969277 | *Paphia textile* | 1 | 0.0282 | 0.0298 | 1.0568 | 0.0208 | | 0.0062 | 0.0703 | 0.0048 | 0.0213 | 0.0273 | 0.0315 | 0.0066 | 0.0395 | 0.0146 | 0.0479 | 0.0987 |
| JF969276 | *Paphia amabilis* | 1 | 0.0600 | 0.0411 | 0.6860 | 0.0222 | 0.0162 | 0.0598 | 0.0411 | 0.0369 | 0.0672 | 0.0266 | 0.0408 | 0.0270 | 0.0224 | | 0.0693 | 0.1035 |
| GQ903339 | *Meretrix lusoria* | 1 | 0.0654 | 0.0129 | 0.1969 | 0.0370 | | 0.0251 | 0.0961 | 0.0195 | 0.0499 | 0.0222 | 0.0985 | 0.0001 | 0.2169 | 0.1643 | 0.0792 | 0.2989 |
| EF043342 | *Lucinella divaricata* | 1 | 0.0094 | 0.0424 | 4.4955 | 0.1626 | 0.1032 | 0.0053 | 0.0046 | 0.0027 | 0.0159 | 0.0209 | 0.0647 | 0.0040 | 0.0091 | 0.0233 | 0.0041 | 0.2772 |
| EF043341 | *Loripes lacteus* | 1 | 0.0105 | 0.0553 | 5.2461 | 0.0124 | 0.1752 | 0.0001 | 0.0085 | 0.0017 | 0.0102 | 0.0817 | 0.0926 | 0.0099 | 0.0096 | 0.0178 | 0.0009 | 0.0389 |
| DQ632743 | *Acanthocardia tuberculata* | 1 | 0.0481 | 0.1819 | 3.7826 | 0.0097 | | 0.0208 | 0.0074 | 0.0157 | 0.0211 | 0.0478 | 0.1627 | 0.0234 | 0.0751 | 0.4939 | 0.1067 | 0.2459 |
| JN398367 | *Solecurtus divaricatus* | 1 | 0.0169 | 0.0705 | 4.1617 | 0.0202 | | 0.0128 | 0.0206 | 0.0990 | 0.0422 | 0.0579 | 0.0764 | 0.0308 | 0.0198 | 0.0445 | 0.0022 | 0.0400 |
| JN398365 | *Semele scabra* | 1 | 0.0185 | 0.1184 | 6.4032 | 0.0081 | | 0.0075 | 0.0157 | 0.0167 | 0.0342 | 0.0598 | 0.0711 | 0.0082 | 0.0214 | 0.0453 | 0.0069 | 0.0745 |
| JN398364 | *Nuttallia olivacea* | 1 | 0.0176 | 0.1339 | 7.6300 | 0.0559 | | 0.0121 | 0.0101 | 0.0112 | 0.0253 | 0.0400 | 0.0916 | 0.0129 | 0.0201 | 0.0359 | 0.0128 | 0.0258 |
| JN398363 | *Soletellina diphos* | 1 | 0.0134 | 0.0723 | 5.3986 | 0.0137 | | 0.0087 | 0.0359 | 0.0905 | 0.0197 | 0.0293 | 0.0881 | 0.1082 | 0.0122 | 0.0265 | 0.0167 | 0.0341 |
| JN398362 | *Moerella iridescens* | 1 | 0.0151 | 0.0839 | 5.5424 | 0.0241 | | 0.0061 | 0.0096 | 0.0031 | 0.0070 | 0.0229 | 0.0085 | 0.0346 | 0.0259 | 0.0015 | 0.0233 | 0.0728 |
| KC832317 | *Meretrix lyrata* | 1 | 0.0573 | 0.0541 | 0.9438 | | 0.0072 | 0.0473 | 0.0760 | 0.1010 | 0.0476 | 0.0259 | 0.1848 | 0.0084 | 0.0438 | 0.0793 | 0.0350 | 0.0995 |
| AY365193 | *Lampsilis ornata* | 1 | 0.0677 | 0.0324 | 0.4784 | 0.0772 | 0.0250 | 0.0073 | 0.0530 | 0.0240 | 0.0644 | 0.0472 | 0.0958 | 0.1727 | 0.0932 | 0.1196 | 0.0618 | 0.1528 |
| HM347668 | *Hyriopsis cumingii* | 1 | 0.0596 | 0.0126 | 0.2115 | 0.1685 | 0.1741 | 0.0001 | 0.0234 | 0.0119 | 0.0430 | 0.1631 | 0.1192 | 0.0211 | 0.0873 | | 0.0698 | 0.0650 |
| KC848654 | *Solenaia carinatus* | 1 | 0.0542 | 0.0251 | 0.4624 | 0.0621 | 0.1783 | 0.0055 | 0.0319 | 0.0445 | 0.0706 | 0.0395 | 0.1073 | 0.1692 | 0.0374 | 0.0356 | 0.0602 | 0.0910 |
| HM856639 | *Toxolasma parvus* | 1 | 0.0778 | 0.0417 | 0.5363 | 0.0809 | 0.0135 | 0.0134 | 0.0401 | 0.0232 | 0.0525 | 0.0456 | 0.1014 | 0.0920 | 0.0948 | 0.0581 | 0.0899 | 0.1081 |
| HM856638 | *Lasmigona compressa* | 1 | 0.0534 | 0.0257 | 0.4814 | 0.0366 | 0.0566 | 0.0173 | 0.0636 | 0.0442 | 0.0414 | 0.0676 | 0.0589 | | 0.0682 | 0.1093 | 0.0765 | 0.0627 |
| HM856637 | *Utterbackia imbecillis* | 1 | 0.0754 | 0.0380 | 0.5039 | 0.1575 | | 0.0081 | 0.0267 | 0.0421 | 0.1127 | 0.0677 | 0.0885 | 0.0812 | 0.0905 | 0.1471 | 0.1252 | 0.1197 |
| HM856635 | *Utterbackia peninsularis* | 1 | 0.0496 | 0.2065 | 4.1630 | 0.0177 | 0.0107 | 0.0191 | 0.0416 | 0.0020 | 0.1196 | 0.0822 | 0.1027 | 0.1512 | 0.0209 | 0.3393 | 0.0371 | 0.0389 |
| HM856634 | *Margaritifera falcata* | 1 | 0.0510 | 0.0200 | 0.3914 | 0.0492 | 0.0600 | 0.0089 | 0.0096 | 0.0300 | 0.0466 | 0.0330 | 0.1461 | 0.1025 | 0.0592 | 0.0001 | | 0.0449 |
| HM014130 | *Unio pictorum* | 1 | 0.0561 | 0.0313 | 0.5570 | 0.0910 | 0.1912 | 0.0092 | 0.0333 | 0.0171 | 0.0524 | 0.0699 | 0.0522 | 0.0714 | 0.0500 | 0.1272 | 0.0630 | 0.1158 |

| | | | | | | | | | | | | | | | | | | |
|---|---|---|---|---|---|---|---|---|---|---|---|---|---|---|---|---|---|---|
| FJ809754 | *Pyganodon grandis* | 1 | 0.0550 | 0.0295 | 0.5357 | 0.1014 | 0.0001 | 0.0068 | 0.0499 | 0.0162 | 0.0914 | 0.0448 | 0.0696 | 0.0410 | 0.0621 | 0.0245 | 0.0668 | 0.0915 |
| FJ809752 | *Venustaconcha ellipsiformis* | 1 | 0.0453 | 0.2116 | 4.6727 | 0.0141 | 0.1120 | 0.0101 | 0.0309 | 0.0197 | 0.1933 | 0.1625 | 0.4402 | 0.1451 | 0.0178 | 0.1303 | 0.0277 | 0.2511 |
| FJ809750 | *Quadrula quadrula* | 1 | 0.0981 | 0.0641 | 0.6531 | 0.0653 | | 0.0161 | 0.0495 | 0.0576 | 0.0613 | 0.1039 | 0.0935 | 0.0494 | 0.1406 | 1.3450 | 0.0932 | 0.0603 |
| JQ728447 | *Solemya velum* | 1 | 0.0461 | 0.2272 | 4.9319 | 0.0600 | 0.5677 | 0.0309 | 0.0796 | 0.0509 | 0.0300 | 0.0986 | 0.0847 | 0.1014 | 0.0325 | 0.0046 | 0.1084 | 0.0094 |
| KJ607173 | *Tegillarca granosa* | 1 | 0.0557 | 0.1294 | 2.3212 | 0.0259 | | 0.0083 | 0.1137 | 0.0185 | 0.0223 | 0.0848 | 0.0952 | 0.0160 | 0.0288 | 0.0179 | 0.0156 | 0.0288 |
| KF750628 | *Scapharca kagoshimensis* | 1 | 0.1512 | 0.0950 | 0.6282 | 0.0138 | | 0.0568 | | 0.1299 | 0.0732 | 0.0890 | | 0.2101 | 0.0305 | 0.0004 | 0.2443 | 2.0675 |
| JX050180 | *Lamprotula coreana* | 1 | 0.0437 | 0.0201 | 0.4598 | 0.0388 | 0.3999 | 0.0201 | 0.0273 | 0.0089 | 0.0425 | 0.0232 | 0.0590 | 0.0364 | 0.0688 | 0.1155 | 0.0419 | 0.0608 |
| KM657954 | *Unio douglasiae* | 1 | 0.0519 | 0.0295 | 0.5694 | 0.0713 | 0.0782 | 0.0085 | 0.0027 | 0.0087 | 0.0712 | 0.0250 | 0.0597 | 0.0245 | 0.0608 | 0.0354 | 0.0560 | 0.0873 |
| KJ754823 | *Mactra chinensis* | 1 | 0.0128 | 0.0565 | 4.3979 | 0.0025 | | 0.0010 | 0.0316 | 0.0029 | 0.0167 | 0.0313 | 0.0582 | 0.0054 | 0.0038 | 0.0112 | 0.0269 | 0.0523 |
| KM580067 | *Panopea generosa* | 1 | 0.0095 | 0.0503 | 5.3072 | 0.0232 | 0.0001 | 0.0030 | 0.1959 | 0.0005 | 0.0399 | 0.0289 | 0.0491 | 0.0068 | 0.0294 | 0.0013 | 0.0071 | 0.0371 |
| KM580068 | *Panopea globosa* | 1 | 0.0080 | 0.0457 | 5.7076 | 0.0039 | | 0.0011 | 0.0160 | 0.0024 | 0.0319 | 0.0295 | 0.0961 | 0.0087 | 0.0348 | 0.1062 | 0.0148 | 0.0591 |
| KM233636 | *Brachidontes exustus* | 1 | 0.0219 | 0.0881 | 4.0152 | 0.0754 | 0.5735 | 0.0235 | 0.0788 | 0.0220 | 0.0040 | 0.0068 | 0.0441 | 0.0176 | 0.0711 | 0.0295 | 0.0391 | 0.1329 |
| KJ577549 | *Mytilus coruscus* | 1 | 0.0403 | 0.0272 | 0.6748 | 0.0053 | | 0.0023 | 0.0033 | 0.0074 | 0.0085 | 0.0196 | 0.0465 | 0.0121 | 0.1955 | 0.0145 | 0.0566 | 0.0265 |
| KJ755996 | *Mya arenaria* | 1 | 0.0905 | 0.4006 | 4.4287 | 0.1179 | | 0.0732 | 0.0445 | 0.1039 | 0.1126 | 0.0038 | 0.2250 | 0.1928 | 0.0663 | 0.0367 | 0.0564 | 0.4209 |
| KF514426 | *Dahurinaia dahurica* | 1 | 0.0421 | 0.0177 | 0.4201 | 0.0254 | 0.0001 | 0.0060 | 0.0331 | 0.0082 | 0.0365 | 0.0353 | 0.0514 | 0.0081 | 0.0602 | 0.0790 | 0.0239 | 0.1727 |
| KJ144818 | *Arconaia lanceolata* | 1 | 0.2291 | 0.0377 | 0.1645 | 0.0703 | 0.0001 | 0.0001 | 0.0307 | 0.0001 | 0.8151 | | 0.0807 | 0.3928 | 0.0565 | 0.0655 | 0.2054 | 0.0001 |
| KJ018924 | *Lamprotula gottschei* | 1 | 0.0759 | 0.0432 | 0.5690 | 0.0545 | 0.0143 | 0.0223 | 0.0339 | 0.0152 | 0.0494 | 0.0422 | 0.1536 | 0.1292 | 0.0602 | 0.0770 | 0.1049 | 0.0647 |
| JQ691662 | *Lamprotula leai* | 1 | 0.0737 | 0.0439 | 0.5958 | 0.0950 | | 0.0115 | 0.0446 | 0.0140 | 0.0613 | 0.0660 | 0.0876 | 0.1247 | 0.0944 | 0.1093 | 0.0763 | 0.1242 |
| HG799089 | *Lutraria rhynchaena* | 1 | 0.0278 | 0.1406 | 5.0631 | 0.1280 | | 0.0081 | 0.0222 | 0.0312 | 0.0306 | 0.0820 | 0.0507 | 0.0316 | 0.0172 | 0.1027 | 0.0447 | 0.1188 |
| KF601246 | *Argopecten purpuratus* | 1 | 0.0650 | 0.0171 | 0.2627 | 0.0486 | | 0.0032 | 0.0142 | 0.0001 | 0.0241 | 0.0355 | 0.0321 | | 0.0441 | | 0.0113 | 0.0437 |
| KP967577 | *Saccostrea cucullata* | 1 | 0.0271 | 0.0372 | 1.3728 | 0.0200 | | 0.0001 | 0.0068 | 0.0246 | 0.0280 | 0.0173 | 0.0539 | 0.0042 | 0.0190 | 0.0114 | 0.0512 | 0.0440 |
| KR856227 | *Crassostrea gasar* | 1 | 0.0329 | 0.0643 | 1.9524 | 0.0186 | 0.2727 | 0.0085 | 0.0087 | 0.0078 | 0.0089 | 0.0028 | 0.0332 | 0.0985 | 0.0310 | 0.0147 | 0.0406 | 0.0960 |
| KF667530 | *Anodonta arcaeformis* | 1 | 0.0766 | 0.0039 | 0.0506 | 0.1123 | | 0.0145 | 0.0001 | 0.0001 | 0.0442 | 0.1493 | 0.1044 | 0.1981 | 0.1005 | 0.3934 | 0.0886 | 0.0330 |
| KF667529 | *Anodonta lucida* | 1 | 0.0819 | 0.0473 | 0.5767 | 0.1482 | 0.0979 | 0.0110 | 0.0603 | 0.0380 | 0.0580 | 0.0938 | 0.1221 | 0.1266 | 0.0470 | 0.0760 | 0.1020 | 0.1669 |

| | | | | | | | | | | | | | | | | | | |
|---|---|---|---|---|---|---|---|---|---|---|---|---|---|---|---|---|---|---|
| KM655841 | *Perna perna* | 1 | 0.0166 | 0.0824 | 4.9615 | 0.0487 | | 0.0242 | 0.1074 | 0.0449 | 0.0019 | 0.0219 | 0.0074 | 0.0018 | 0.0359 | 0.0596 | 0.0139 | 0.0264 |
| KP205428 | *Tridacna squamosa* | 1 | 0.1043 | 0.9115 | 8.7391 | 0.0054 | | 0.0058 | 0.0142 | 0.0487 | 0.0218 | 0.3568 | 0.3273 | 0.5336 | 0.1362 | 0.7820 | 0.0161 | |
| KP954700 | *Anadara vellicata* | 1 | 0.0429 | 0.0681 | 1.5876 | | | 0.0106 | 0.0084 | 0.0065 | 0.0364 | 0.0001 | | 0.0440 | 0.0511 | 0.0225 | 0.0090 | 0.0510 |
| KU975161 | *Trisidos kiyoni* | 1 | 0.0412 | 0.2676 | 6.4931 | 0.0231 | | 0.0156 | 0.0016 | 0.0572 | 0.0250 | 0.1005 | 0.1280 | 0.0062 | 0.0889 | 0.0322 | 0.0205 | 0.0199 |
| KU975162 | *Potiarca pilula* | 1 | 0.0583 | 0.1416 | 2.4294 | 0.0507 | | 0.0256 | 0.0533 | 0.0213 | 0.0442 | 0.0603 | 0.0050 | 0.0426 | 0.0469 | 0.1286 | 0.0124 | 0.1027 |
| KP187851 | *Anodonta euscaphys* | 1 | 0.1643 | 0.0079 | 0.0481 | 0.1726 | 0.0735 | 0.0891 | | 0.1114 | 0.1095 | 0.0805 | 0.3326 | 0.0001 | 0.4194 | | 0.1384 | 0.2573 |
| KT247374 | *Potomida littoralis* | 1 | 0.0672 | 0.0340 | 0.5051 | 0.0670 | 0.0001 | 0.0033 | 0.0392 | 0.0309 | 0.0593 | 0.0528 | 0.1683 | 0.0740 | 0.0505 | 0.0197 | 0.0936 | 0.1405 |
| KT723012 | *Leptodea leptodon* | 1 | 0.0632 | 0.0224 | 0.3549 | 0.1152 | 0.0001 | 0.0070 | 0.0851 | 0.0482 | 0.0494 | 0.0374 | 0.0991 | 0.0826 | 0.0398 | 0.0001 | 0.0818 | 0.1560 |
| KJ495725 | *Lanceolaria grayana* | 1 | 0.0988 | 0.0091 | 0.0916 | 0.0487 | | 0.0058 | 0.0878 | 0.0951 | | 0.0928 | 0.1036 | 0.0021 | 0.2460 | 0.1462 | 0.1855 | 0.0000 |
| KP273584 | *Cuneopsis pisciculus* | 1 | 0.0559 | 0.0266 | 0.4762 | 0.1022 | 0.1009 | 0.0001 | 0.0526 | 0.0084 | 0.0317 | 0.0675 | 0.0974 | 0.0157 | 0.0501 | 0.0874 | 0.0654 | 0.1336 |
| KP419933 | *Saxidomus purpuratus* | 1 | 0.0239 | 0.1295 | 5.4182 | 0.0216 | | 0.0169 | 0.0567 | 0.0204 | 0.0452 | 0.0318 | 0.1331 | 0.0966 | 0.0174 | 0.0028 | 0.0184 | 0.1631 |
| KR862368 | *Calyptogena magnifica* | 1 | 0.0650 | 0.2790 | 4.2939 | 0.1005 | 0.0803 | 0.0383 | 0.0891 | 0.0394 | 0.0681 | 0.0818 | 0.1309 | 0.0365 | 0.0207 | 0.0513 | 0.1166 | 0.1257 |
| KT161261 | *Argopecten ventricosus* | 1 | 0.0310 | 0.0076 | 0.2454 | 0.0085 | | 0.0076 | 0.0153 | 0.0119 | 0.0384 | 0.0102 | 0.2509 | 0.0254 | 0.0107 | 0.0001 | 0.0430 | 0.3691 |
| KP756905 | *Limnoperna fortunei* | 1 | 0.0402 | 0.2612 | 6.4916 | 0.0499 | 0.2005 | 0.0286 | 0.0661 | 0.0549 | 0.0625 | 0.0208 | 0.0355 | 0.0126 | 0.0264 | 0.3734 | 0.0499 | 0.1428 |
| AY588938 | *Haliotis rubra* | 2 | 0.0176 | 0.0033 | 0.1899 | 0.0264 | | 0.0121 | 0.0241 | 0.0001 | 0.0045 | 0.0706 | 0.0296 | 0.0648 | 0.0001 | 0.0538 | 0.0090 | 0.0001 |
| KJ472483 | *Haliotis laevigata* | 2 | 0.0264 | 0.0057 | 0.2162 | 0.0136 | 0.0001 | 0.0001 | 0.0001 | 0.0001 | 0.0001 | 0.0212 | 0.0430 | 0.0300 | 0.0115 | 0.0001 | 0.0189 | 0.0110 |
| KF724723 | *Haliotis discus hannai* | 2 | 0.0214 | 0.0128 | 0.5990 | 0.0173 | 0.0043 | 0.0001 | 0.0156 | 0.0663 | 0.0156 | 0.0075 | 0.0732 | 0.0094 | 0.0183 | 0.0614 | 0.0165 | 0.0091 |
| HQ832672 | *Haliotis diversicolor* | 2 | 0.0285 | 0.0173 | 0.6073 | 0.0206 | 0.0408 | 0.0017 | 0.0096 | 0.0083 | 0.0046 | 0.0499 | 0.0814 | 0.0279 | 0.0324 | 0.0444 | 0.0388 | 0.0430 |
| FJ605487 | *Haliotis tuberculata* | 2 | 0.0303 | 0.0073 | 0.2421 | 0.0204 | 0.0856 | 0.0137 | 0.0301 | 0.0227 | 0.0034 | 0.0676 | 0.0676 | 0.0001 | 0.0222 | 0.0001 | 0.0365 | 0.1412 |
| FJ599667 | *Haliotis tuberculata tuberculata* | 2 | 0.0325 | 0.0063 | 0.1944 | 0.0398 | 0.0001 | 0.0060 | 0.0001 | 0.0001 | 0.0580 | 0.0372 | 0.1227 | 0.0567 | 0.0155 | 0.0001 | 0.0199 | 0.0302 |
| KT207826 | *Tegula lividomaculata* | 2 | 0.0271 | 0.0305 | 1.1235 | 0.0019 | 0.0001 | 0.0010 | 0.0284 | 0.0124 | 0.0145 | 0.0118 | 0.0335 | 0.0166 | 0.0125 | 0.0300 | 0.0133 | 0.0388 |
| JN790613 | *Tegula brunnea* | 2 | 0.0219 | 0.0275 | 1.2550 | 0.0028 | | 0.0023 | 0.0123 | 0.0254 | 0.0038 | 0.0069 | 0.0216 | 0.0674 | 0.0103 | 0.0103 | 0.0116 | 0.1442 |
| KT207824 | *Bolma rugosa* | 2 | 0.0106 | 0.0352 | 3.3135 | 0.0122 | | 0.0001 | 0.0001 | 0.0077 | 0.0087 | 0.0130 | 0.0615 | 0.0474 | 0.0111 | 0.0080 | 0.0072 | 0.0090 |
| KR297248 | *Angaria neglecta* | 2 | 0.0320 | 0.0919 | 2.8676 | 0.0298 | 0.0371 | 0.0250 | 0.0864 | 0.0194 | 0.0223 | 0.0243 | 0.0928 | 0.1125 | 0.0366 | 0.1816 | 0.0302 | 0.0301 |

| | | | | | | | | | | | | | | | | | | |
|---|---|---|---|---|---|---|---|---|---|---|---|---|---|---|---|---|---|---|
| KF700096 | *Lunella aff. cinerea* | 2 | 0.0233 | 0.0560 | 2.4009 | 0.0159 | 0.0017 | 0.0013 | 0.0131 | 0.0101 | 0.0112 | 0.0130 | 0.0797 | 0.0304 | 0.0281 | 0.0726 | 0.0278 | 0.0343 |
| LC107880 | *Lepetodrilus nux* | 2 | 0.0265 | 0.0130 | 0.4924 | 0.0145 | 0.0746 | 0.0001 | 0.1105 | 0.0726 | 0.0019 | 0.0109 | 0.0599 | 0.0281 | 0.0081 | 0.0052 | 0.0348 | 0.0954 |
| KR297250 | *Lepetodrilus schrolli* | 2 | 0.0256 | 0.0105 | 0.4123 | 0.0041 | 0.0000 | 0.0050 | 0.0001 | 0.0001 | 0.0747 | 0.0146 | 0.0407 | 0.0556 | 0.0301 | 0.0000 | 0.0641 | 0.1321 |
| KR297249 | *Granata lyrata* | 2 | 0.0648 | 0.1766 | 2.7250 | 0.3102 | 0.3240 | 0.0576 | 0.3394 | 0.2137 | 0.0418 | 0.1276 | 0.0997 | 0.0281 | 0.0481 | 0.0808 | 0.0950 | 0.0357 |
| KT207825 | *Diodora graeca* | 2 | 0.0398 | 0.0969 | 2.4328 | 0.0327 | 0.1067 | 0.0294 | 0.0451 | 0.0410 | 0.0207 | 0.0470 | 0.0911 | 0.0013 | 0.0307 | 0.1205 | 0.1100 | 0.0416 |
| JN790612 | *Fissurella volcano* | 2 | 0.0420 | 0.1134 | 2.7031 | 0.0518 | 0.3732 | 0.0169 | 0.0497 | 0.0425 | 0.0255 | 0.0515 | 0.0325 | 0.0579 | 0.0561 | 0.4582 | 0.0376 | 0.0387 |
| AP013032 | *Chrysomallon squamiferum* | 2 | 0.0524 | 0.1977 | 3.7773 | 0.0289 | 0.4727 | 0.0539 | 0.0014 | 0.0087 | 0.0304 | 0.0410 | 0.0809 | 0.0387 | 0.0627 | 0.0943 | 0.0360 | 0.0710 |
| KR297251 | *Phasianella solida* | 2 | 0.0652 | 0.1566 | 2.4030 | 0.0424 | 0.0226 | 0.0260 | 0.1035 | 0.0551 | 0.0397 | 0.0440 | 0.1433 | 0.0823 | 0.0811 | 0.1158 | 0.0524 | 0.1556 |
| KF728890 | *Nerita versicolor* | 2 | 0.0146 | 0.0083 | 0.5675 | 0.0091 | 0.0673 | 0.0027 | 0.0001 | 0.0062 | 0.0032 | 0.0168 | 0.0235 | 0.0105 | 0.0135 | 0.0001 | 0.0135 | 0.0344 |
| GU810158 | *Nerita melanotragus* | 2 | 0.0134 | 0.0103 | 0.7677 | 0.0090 | 0.1872 | 0.0039 | 0.0147 | 0.0275 | 0.0135 | 0.0151 | 0.0136 | 0.0057 | 0.0220 | 0.0001 | 0.0123 | 0.0138 |
| KF728889 | *Nerita tessellata* | 2 | 0.0242 | 0.0068 | 0.2813 | 0.0001 | 0.0000 | 0.0064 | 0.1347 | 0.0270 | 0.0166 | 0.0210 | 0.0099 | 0.0001 | 0.0044 | 0.0001 | 0.0218 | 0.0001 |
| KF728888 | *Nerita fulgurans* | 2 | 0.0140 | 0.0033 | 0.2339 | 0.0186 | 0.0000 | 0.0001 | 0.0001 | 0.0400 | 0.0090 | 0.0075 | 0.0151 | 0.0305 | 0.0339 | 0.0207 | 0.0138 | 0.0194 |
| KF148053 | *Aplysia kurodai* | 2 | 0.0021 | 0.0122 | 5.7484 | 0.1421 | 0.0001 | 0.0022 | | 0.0193 | 0.0064 | 0.0467 | 0.0082 | 0.0844 | 0.0115 | 0.0000 | 0.0109 | 0.0170 |
| AY569552 | *Aplysia californica* | 2 | 0.0247 | 0.0164 | 0.6652 | 0.0314 | 0.0001 | 0.0358 | 0.0193 | 0.0015 | 0.0163 | 0.1010 | 0.0110 | 0.0321 | 0.0030 | 0.0001 | 0.0270 | 0.0048 |
| DQ991927 | *Aplysia dactylomela* | 2 | 0.0159 | 0.0074 | 0.4646 | 0.0031 | 0.0813 | 0.0001 | 0.0001 | 0.0046 | 0.0073 | 0.0782 | 0.0091 | 0.0644 | 0.0025 | 0.0088 | 0.0095 | 0.0171 |
| AY083457 | *Roboastra europaea* | 2 | 0.0222 | 0.0859 | 3.8690 | 0.0295 | 0.0233 | 0.0074 | 0.0359 | 0.0234 | 0.0283 | 0.0310 | 0.0194 | 0.0801 | 0.0323 | 0.0163 | 0.0156 | 0.0926 |
| KP764764 | *Tritonia diomedea* | 2 | 0.0145 | 0.0652 | 4.4814 | 0.0503 | | 0.0069 | 0.0134 | 0.0161 | 0.0184 | 0.0118 | 0.0245 | 0.0421 | 0.0167 | 0.0045 | 0.0076 | 0.0033 |
| JN620539 | *Melibe leonina* | 2 | 0.0262 | 0.0853 | 3.2520 | 0.0417 | 0.0001 | 0.0111 | 0.0186 | 0.0296 | 0.0263 | 0.0256 | 0.0695 | 0.0151 | 0.0162 | 0.0080 | 0.0315 | 0.0246 |
| JN627205 | *Salinator rhamphidia* | 2 | 0.0343 | 0.1150 | 3.3562 | 0.0409 | 0.5129 | 0.0150 | 0.0396 | 0.0275 | 0.0230 | 0.0172 | 0.0560 | 0.0344 | 0.0341 | 0.0028 | 0.0278 | 0.0451 |
| AY345049 | *Siphonaria gigas* | 2 | 0.0399 | 0.1275 | 3.1995 | 0.0195 | 0.2108 | 0.0168 | 0.0632 | 0.0265 | 0.0243 | 0.0198 | 0.0533 | 0.0489 | 0.0301 | 0.1057 | 0.0259 | 0.1330 |
| AY345022 | *Siphonaria pectinata* | 2 | 0.0129 | 0.0870 | 6.7172 | 0.0113 | 0.3578 | 0.0047 | 0.0171 | 0.0132 | 0.0133 | 0.0103 | 0.0319 | 0.0206 | 0.0250 | 0.0135 | 0.0186 | 0.0109 |
| EU599581 | *Ascobulla fragilis* | 2 | 0.0218 | 0.0989 | 4.5385 | 0.0616 | 0.0001 | 0.0119 | 0.0219 | 0.0224 | 0.0238 | 0.0147 | 0.0200 | 0.0272 | 0.0266 | 0.0934 | 0.0314 | 0.0212 |
| AY345048 | *Elysia chlorotica* | 2 | 0.0420 | 0.1422 | 3.3861 | 0.0714 | 0.5180 | 0.0116 | 0.0270 | 0.0238 | 0.0462 | 0.0227 | 0.0889 | 0.0557 | 0.0662 | 0.0387 | 0.0689 | 0.0131 |
| JN632509 | *Onchidella celtica* | 2 | 0.0330 | 0.1005 | 3.0401 | 0.0468 | | 0.0141 | 0.0320 | 0.0287 | 0.0162 | 0.0266 | 0.0681 | 0.0394 | 0.0292 | 0.0934 | 0.0487 | 0.0612 |

| | | | | | | | | | | | | | | | | | | |
|---|---|---|---|---|---|---|---|---|---|---|---|---|---|---|---|---|---|---|
| JN632509 | *Trimusculus reticulatus* | 2 | 0.0252 | 0.0858 | 3.4001 | 0.0337 | 0.0034 | 0.0029 | 0.0158 | 0.0070 | 0.0318 | 0.0137 | 0.0252 | 0.0381 | 0.0349 | 0.0426 | 0.0390 | 0.0418 |
| AY345054 | *Pyramidella dolabrata* | 2 | 0.0559 | 0.1996 | 3.5695 | 0.0462 | | 0.0383 | 0.0398 | 0.0680 | 0.0439 | 0.0390 | 0.0563 | 0.0165 | 0.0747 | 0.0459 | 0.1135 | 0.1859 |
| AB028237 | *Pupa strigosa* | 2 | 0.0509 | 0.1677 | 3.2946 | 0.0576 | 0.6721 | 0.0142 | 0.0607 | 0.0392 | 0.0392 | 0.0311 | 0.1004 | 0.0833 | 0.0333 | 0.1128 | 0.0676 | 0.0736 |
| DQ991933 | *Micromelo undata* | 2 | 0.0143 | 0.0723 | 5.0582 | 0.0295 | | 0.0065 | 0.0114 | 0.0468 | 0.0141 | 0.0004 | 0.0115 | 0.0584 | 0.0265 | 0.0116 | 0.0227 | 0.0234 |
| KP716634 | *Naticarius hebraeus* | 2 | 0.0154 | 0.0665 | 4.3168 | 0.0266 | 0.0143 | 0.0070 | 0.0121 | 0.0371 | 0.0109 | 0.0153 | 0.0182 | | 0.0187 | | 0.0149 | 0.0174 |
| KM245630 | *Strombus gigas* | 2 | 0.0246 | 0.0730 | 2.9715 | 0.0110 | 0.0019 | 0.0052 | 0.0215 | 0.0131 | 0.0135 | 0.0205 | 0.0357 | 0.0573 | 0.0366 | 0.0479 | 0.0205 | 0.0382 |
| KP716635 | *Galeodea echinophora* | 2 | 0.0107 | 0.0371 | 3.4842 | 0.0217 | | 0.0096 | 0.0158 | 0.0091 | 0.0127 | 0.0130 | 0.0016 | 0.0243 | 0.0127 | 0.0042 | 0.0051 | 0.0523 |
| EU827200 | *Cymatium parthenopeum* | 2 | 0.0132 | 0.0405 | 3.0814 | 0.0194 | | 0.0033 | 0.0037 | 0.0145 | 0.0138 | 0.0146 | 0.0273 | 0.0088 | 0.0102 | 0.0223 | 0.0224 | 0.0327 |
| HM174254 | *Eualetes tulipa* | 2 | 0.0472 | 0.1539 | 3.2626 | 0.0275 | 0.0025 | 0.0122 | 0.0747 | 0.0225 | 0.0215 | 0.0283 | 0.0253 | 0.0301 | 0.0763 | 0.0644 | 0.0744 | 0.0831 |
| HM174253 | *Dendropoma maximum* | 2 | 0.0429 | 0.1340 | 3.1244 | 0.0249 | 0.0904 | 0.0042 | 0.0279 | 0.0007 | 0.0156 | 0.0633 | 0.1218 | 0.0218 | 0.0719 | 2.4020 | 0.0650 | 0.0458 |
| HM174252 | *Dendropoma gregarium* | 2 | 0.0569 | 0.1807 | 3.1755 | 0.0318 | 0.0634 | 0.0157 | 0.0427 | 0.0001 | 0.0566 | 0.0524 | 0.0741 | 0.1065 | 0.0944 | 0.0646 | 0.0630 | 0.1697 |
| HM174255 | *Thylacodes squamigerus* | 2 | 0.0495 | 0.1344 | 2.7152 | 0.0276 | 0.2600 | 0.0087 | 0.0620 | 0.0244 | 0.0213 | 0.0295 | 0.0897 | 0.0353 | 0.1046 | 0.0640 | 0.0844 | 0.0839 |
| KT962044 | *Buccinum pemphigus* | 2 | 0.0145 | 0.0072 | 0.4987 | 0.0145 | 0.0784 | 0.0050 | 0.0198 | 0.0340 | 0.0032 | 0.0283 | 0.0143 | 0.0281 | 0.0187 | 0.0001 | 0.0826 | |
| KF897830 | *Babylonia lutosa* | 2 | 0.0746 | 0.0164 | 0.2199 | 0.0100 | 0.0789 | 0.0183 | 0.0001 | 0.0001 | 0.0057 | 0.0841 | | | 0.0063 | 0.0000 | 0.0166 | |
| HQ416443 | *Babylonia areolata* | 2 | 0.0669 | 0.0120 | 0.1790 | 0.0892 | 0.0323 | 0.0000 | 0.0729 | 0.0001 | 0.0315 | 0.0229 | | 0.2010 | | 0.0001 | 0.0624 | |
| KT382829 | *Volutharpa perryi* | 2 | 0.0172 | 0.0051 | 0.2932 | 0.0001 | 0.1818 | 0.0020 | 0.0053 | 0.0052 | 0.0059 | 0.0018 | 0.1619 | 0.0774 | 0.0432 | 0.0000 | 0.0097 | 0.0063 |
| KM603509 | *Varicinassa variciferus* | 2 | 0.0122 | 0.0106 | 0.8709 | 0.0001 | 0.0001 | 0.0118 | 0.0056 | 0.0024 | 0.0149 | 0.0138 | 0.0169 | 0.0100 | 0.0070 | 0.0210 | 0.0131 | 0.0205 |
| EU827201 | *Nassarius reticulatus* | 2 | 0.0126 | 0.0091 | 0.7235 | 0.0171 | 0.0001 | 0.0019 | 0.0022 | | 0.0165 | 0.0040 | 0.0253 | 0.0175 | 0.0038 | 0.0001 | 0.0128 | 0.0335 |
| EU827195 | *Cancellaria cancellata* | 2 | 0.0313 | 0.0971 | 3.1044 | 0.0244 | 0.1435 | 0.0183 | | 0.0323 | 0.0132 | 0.0307 | 0.0528 | 0.0287 | 0.0303 | 0.0442 | 0.0261 | 0.0190 |
| KT199301 | *Conus tribblei* | 2 | 0.0203 | 0.0136 | 0.6687 | 0.0116 | 0.1044 | 0.0062 | 0.0039 | 0.0051 | 0.0135 | 0.0055 | 0.0573 | 0.0338 | 0.0187 | 0.0497 | 0.0329 | 0.0372 |
| KR006970 | *Conus tulipa* | 2 | 0.0204 | 0.0175 | 0.8576 | 0.0184 | 0.0133 | 0.0001 | 0.0073 | 0.0113 | 0.0051 | 0.0086 | 0.0434 | 0.0240 | 0.0422 | 0.0045 | 0.0282 | 0.0508 |
| DQ862058 | *Conus textile* | 2 | 0.0259 | 0.0183 | 0.7072 | 0.0159 | 0.1116 | 0.0013 | 0.0039 | 0.0089 | 0.0109 | 0.0234 | 0.0744 | 0.0385 | 0.0365 | 0.0396 | 0.0190 | 0.0569 |
| KF887950 | *Conus consors* | 2 | 0.0167 | 0.0132 | 0.7876 | 0.0219 | 0.2762 | 0.0027 | 0.0001 | 0.0084 | 0.0046 | 0.0049 | 0.0374 | 0.0084 | 0.0250 | 0.0117 | 0.0168 | 0.0184 |
| EU827198 | *Conus borgesi* | 2 | 0.0449 | 0.0196 | 0.4358 | 0.0604 | 0.0282 | 0.0074 | 0.0136 | 0.0761 | 0.0289 | 0.0283 | 0.0799 | 0.0055 | 0.0528 | 0.1005 | 0.0692 | 0.0540 |

| | | | | | | | | | | | | | | | | | | |
|---|---|---|---|---|---|---|---|---|---|---|---|---|---|---|---|---|---|---|
| EU827197 | *Fusiturris similis* | 2 | 0.0227 | 0.0518 | 2.2818 | 0.0488 | 0.1324 | 0.0142 | 0.0190 | 0.0207 | 0.0237 | 0.0125 | 0.0348 | 0.0739 | 0.0226 | 0.0488 | 0.0342 | 0.0310 |
| DQ284754 | *Lophiotoma cerithiformis* | 2 | 0.0134 | 0.0371 | 2.7631 | 0.0166 | 0.0477 | 0.0093 | 0.0146 | 0.0144 | 0.0023 | 0.0155 | 0.0131 | 0.0217 | 0.0138 | 0.0055 | 0.1429 | 0.0144 |
| EU827196 | *Terebra dimidiata* | 2 | 0.0195 | 0.0549 | 2.8100 | 0.0149 | 0.0129 | 0.0113 | 0.0125 | 0.0069 | 0.0051 | 0.0130 | 0.0342 | 0.0164 | 0.0245 | | 0.0291 | 0.0139 |
| KM213962 | *Rapana venosa* | 2 | 0.0076 | 0.0108 | 1.4314 | 0.0064 | 0.0001 | 0.0001 | 0.0001 | 0.0082 | 0.0031 | 0.0067 | 0.0096 | 0.0468 | 0.0058 | 0.0402 | 0.0106 | 0.0183 |
| JQ446041 | *Concholepas concholepas* | 2 | 0.0696 | 0.1076 | 1.5462 | 0.0125 | 0.0102 | 0.0038 | 0.0018 | 0.5936 | 0.0157 | 0.0285 | 0.3261 | 0.0069 | 0.0078 | 0.0213 | 0.0228 | 0.0229 |
| DQ159954 | *Reishia clavigera* | 2 | 0.0181 | 0.0165 | 0.9085 | 0.0142 | 0.0000 | 0.0001 | 0.0442 | 0.0109 | 0.0072 | 0.0428 | 0.0496 | 0.0072 | 0.0114 | 0.0001 | 0.0119 | 0.0150 |
| EU827194 | *Bolinus brandaris* | 2 | 0.0157 | 0.0464 | 2.9547 | 0.0203 | 0.0968 | 0.0019 | 0.0078 | 0.0122 | 0.0067 | 0.0110 | 0.0265 | 0.0362 | 0.0193 | 0.0187 | 0.0220 | 0.0218 |
| EU827199 | *Cymbium olla* | 2 | 0.0328 | 0.1009 | 3.0778 | 0.0331 | 0.1634 | 0.0102 | 0.0364 | 0.0385 | 0.0277 | 0.0288 | 0.0334 | 0.0566 | 0.0353 | 0.2984 | 0.0396 | 0.0707 |
| GU196685 | *Amalda northlandica* | 2 | 0.0165 | 0.0469 | 2.8367 | 0.0105 | 0.0166 | 0.0076 | 0.0128 | 0.0140 | 0.0165 | 0.0132 | 0.0197 | 0.0100 | 0.0171 | 0.0040 | 0.0270 | 0.0139 |
| KU221394 | *Turritella bacillum* | 2 | 0.0257 | 0.0709 | 2.7585 | 0.0143 | | 0.0031 | 0.0283 | 0.0163 | 0.0265 | 0.0273 | 0.0436 | 0.0873 | 0.0381 | 0.0081 | 0.0221 | 0.0151 |
| KF736848 | *Semisulcospira libertina* | 2 | 0.0171 | 0.0595 | 3.4876 | 0.0112 | 0.5080 | 0.0015 | 0.0079 | 0.0768 | 0.0101 | 0.0007 | 0.0227 | 0.0343 | 0.0165 | 0.0579 | 0.0236 | 0.0629 |
| KC757644 | *Ifremeria nautilei* | 2 | 0.0334 | 0.0856 | 2.5622 | 0.0038 | 0.0787 | 0.0147 | 0.0337 | 0.0525 | 0.0190 | 0.0537 | 0.0626 | 0.0665 | 0.0457 | 0.0361 | 0.0338 | 0.0273 |
| DQ238599 | *Lottia digitalis* | 2 | 0.2091 | 0.6499 | 3.1087 | 0.2575 | 0.2712 | 0.1423 | 0.3115 | 0.5720 | 0.1460 | 0.1507 | 0.2453 | 0.1121 | 0.2850 | 0.2203 | 0.2316 | 0.3496 |
| JN564796 | *Galba pervia* | 2 | 0.0028 | 0.0295 | 10.5760 | 0.0232 | 0.7279 | 0.0035 | 0.0275 | 0.0209 | 0.0038 | 0.0316 | 0.0026 | 0.0153 | 0.0063 | 0.0834 | 0.0317 | 0.0529 |
| KP098541 | *Radix balthica* | 2 | 0.0099 | 0.0565 | 5.7304 | 0.0261 | 0.0018 | 0.0016 | 0.0554 | 0.0013 | 0.0191 | 0.0014 | 0.0162 | 0.0154 | 0.0092 | 0.2399 | 0.0109 | 0.0542 |
| KP098540 | *Radix auricularia* | 2 | 0.0079 | 0.0483 | 6.0774 | 0.0552 | 0.1504 | 0.0030 | 0.0345 | 0.0018 | 0.0162 | 0.0188 | 0.0095 | 0.0790 | 0.0040 | 0.0848 | 0.0895 | 0.0143 |
| KP279638 | *Radix swinhoei* | 2 | 0.0539 | 0.0161 | 0.2982 | 0.0347 | 0.0001 | 0.0029 | 0.0744 | 0.2716 | 0.0266 | 0.0758 | 0.0967 | 0.0001 | 0.0422 | 0.0479 | 0.0158 | 0.2097 |
| JQ390526 | *Physella acuta* | 2 | 0.1404 | 0.0192 | 0.1365 | | 0.0000 | 0.0000 | 0.0666 | 0.0382 | 0.1004 | 0.1364 | | 0.1584 | | 0.7189 | | 0.0708 |
| KP279639 | *Planorbarius corneus* | 2 | 0.0159 | 0.1078 | 6.7784 | 0.0373 | 0.0001 | 0.0041 | 0.0154 | 0.0244 | 0.0198 | 0.0064 | 0.1290 | 0.0993 | 0.0245 | 0.0547 | 0.0310 | 0.0462 |
| AY380567 | *Biomphalaria glabrata* | 2 | 0.0409 | 0.0283 | 0.6927 | 0.0030 | 0.0000 | 0.0007 | 0.0226 | 0.0104 | 0.0095 | | 0.0900 | 0.0503 | 0.0583 | 0.0323 | 0.0960 | 0.0579 |
| EF433576 | *Biomphalaria tenagophila* | 2 | 0.0363 | 0.0298 | 0.8192 | 0.0022 | 0.0001 | | | 0.0177 | | 0.0122 | 0.0671 | 0.1516 | 0.0819 | | 0.0210 | |
| KM503121 | *Cipangopaliduna cathayensis* | 2 | 0.0372 | 0.1739 | 4.6744 | 0.0086 | 0.2096 | 0.0161 | 0.0692 | 0.0318 | 0.0400 | 0.0363 | 0.0468 | 0.0511 | 0.0425 | 0.1079 | 0.0215 | 0.0597 |
| KM100140 | *Marisa cornuarietis* | 2 | 0.0561 | 0.0518 | 0.9234 | 0.5505 | 0.3996 | 0.0073 | 0.0484 | 0.0466 | 0.0529 | 0.0425 | 0.1419 | 0.0318 | 0.0419 | 0.0065 | 0.0223 | 0.0186 |
| KJ739609 | *Pomacea canaliculata* | 2 | 0.0442 | 0.0115 | 0.2600 | 0.0303 | 0.3276 | 0.0070 | 0.0258 | 0.0074 | 0.0204 | 0.0455 | 0.0883 | | 0.0865 | 0.0413 | 0.4199 | |

| | | | | | | | | | | | | | | | | | | |
|---|---|---|---|---|---|---|---|---|---|---|---|---|---|---|---|---|---|---|
| KR350466 | *Pomacea maculata* | 2 | 0.0334 | 0.0133 | 0.3985 | 0.0392 | 0.0001 | 0.0052 | 0.0254 | 0.0074 | 0.0763 | 0.0198 | 0.0551 | 0.0013 | 0.0369 | 0.0100 | 0.0325 | 0.0140 |
| EU079378 | *Oncomelania hupensis robertsoni* | 2 | 0.0249 | 0.0093 | 0.3746 | 0.0771 | 0.0620 | 0.0001 | 0.0258 | 0.0100 | 0.0071 | 0.0305 | 0.0431 | 0.0641 | 0.0241 | 0.1918 | 0.0498 | |
| EU001660 | *Oncomelania hupensis hupensis* | 2 | 0.0132 | 0.0012 | 0.0876 | 0.0230 | 0.0000 | 0.0001 | 0.0001 | 0.0000 | 0.0001 | 0.0001 | 0.0576 | 0.0000 | 0.0200 | 0.0001 | 0.0257 | 0.0432 |
| FJ997214 | *Oncomelania hupensis* | 2 | 0.0551 | 0.0031 | 0.0561 | 0.0001 | 0.3522 | 0.0267 | | 0.0304 | 0.0001 | 0.0001 | 0.0486 | 0.0001 | 0.0537 | 0.0632 | 0.0419 | 0.2286 |
| EU440735 | *Tricula hortensis* | 2 | 0.0231 | 0.0249 | 1.0765 | 0.0146 | | 0.0097 | 0.0387 | 0.1217 | 0.0258 | 0.0513 | 0.3559 | 0.0269 | 0.0261 | 0.0271 | 0.0144 | 0.0034 |
| GQ996415 | *Potamopyrgus estuarinus* | 2 | 0.0110 | 0.0040 | 0.3637 | 0.0038 | 0.0000 | 0.0094 | | 0.0036 | 0.0075 | 0.0001 | 0.0205 | | 0.0110 | 0.0001 | 0.0123 | |
| GQ996430 | *Potamopyrgus antipodarum* | 2 | 0.0159 | 0.0054 | 0.3404 | 0.0550 | 0.0001 | 0.0001 | 0.0029 | 0.0001 | 0.0050 | 0.0088 | 0.0371 | 0.0225 | 0.0230 | 0.1523 | 0.0119 | 0.0074 |
| KM365085 | *Cerion incanum* | 2 | 0.0680 | 0.2721 | 4.0010 | 0.0544 | 0.4597 | 0.0346 | 0.0532 | 0.0405 | 0.0323 | 0.1188 | 0.1441 | 0.0524 | 0.0796 | 0.1372 | 0.1057 | 0.0064 |
| KJ744205 | *Achatina fulica* | 2 | 0.0808 | 0.2445 | 3.0275 | 0.0458 | 0.0195 | 0.0207 | 0.0881 | 0.0505 | 0.0718 | 0.0615 | 0.0937 | 0.1177 | 0.1150 | 0.3460 | 0.0729 | 0.3938 |
| KM083123 | *Mastigeulota kiangsinensis* | 2 | 0.0261 | 0.1118 | 4.2846 | 0.0313 | 0.1809 | 0.0026 | | 0.0175 | 0.0290 | 0.0181 | 0.0528 | 0.0373 | 0.0343 | 0.0001 | 0.0298 | 0.1712 |
| KR338956 | *Dolicheulota formosensis* | 2 | 0.0241 | 0.1246 | 5.1828 | 0.0553 | | 0.0018 | 0.0114 | 0.0238 | 0.0299 | 0.0153 | 0.0182 | 0.0260 | 0.0146 | 0.0524 | 0.0313 | 0.0799 |
| KT192071 | *Aegista aubryana* | 2 | 0.0517 | 0.0387 | 0.7483 | 0.1667 | 0.0060 | | 0.0176 | 0.0545 | 0.0306 | 0.0145 | 0.0520 | 0.0194 | 0.0559 | 0.0886 | 0.0477 | |
| KR002567 | *Aegista diversifamilia* | 2 | 0.0405 | 0.0495 | 1.2220 | 0.0444 | | 0.0012 | 0.0439 | 0.0283 | 0.0509 | 0.0557 | 0.1695 | 0.0873 | 0.0414 | 0.1120 | 0.0463 | 0.0371 |
| JQ417194 | *Helix aspersa* | 2 | 0.0222 | 0.1473 | 6.6295 | 0.0283 | 0.1195 | 0.0026 | 0.0173 | 0.0214 | 0.0118 | 0.0174 | 0.0347 | 0.0236 | 0.0195 | 7.0330 | 0.0252 | 0.0284 |
| JN107636 | *Cylindrus obtusus* | 2 | 0.0521 | 0.1602 | 3.0776 | 0.1160 | 0.2100 | 0.0104 | 0.0310 | 0.0163 | 0.0405 | 0.0557 | 0.0293 | 0.0714 | 0.0658 | 0.0965 | 0.0587 | 0.1601 |
| KM365408 | *Camaena cicatricosa* | 2 | 0.0309 | 0.1667 | 5.4030 | 0.0318 | | 0.0068 | 0.0234 | 0.0192 | 0.0376 | 0.0196 | 0.0378 | 0.0912 | 0.0235 | 0.1490 | 0.0445 | 0.0001 |
| JN627206 | *Succinea putris* | 2 | 0.0346 | 0.2196 | 6.3444 | 0.0573 | | 0.0150 | 0.0115 | 0.0606 | 0.0297 | 0.0374 | 0.0289 | 0.0653 | 0.0158 | 0.0204 | 0.0338 | 0.0831 |
| KT821554 | *Naesiotus nux* | 2 | 0.0322 | 0.2073 | 6.4437 | 0.0542 | 0.7414 | 0.0114 | 0.0260 | 0.0159 | 0.0360 | 0.0250 | 0.0359 | 0.0785 | 0.0338 | 0.0429 | 0.0315 | 0.2589 |
| NC_001761 | *Albinaria caerulea* | 2 | 0.0320 | 0.1927 | 6.0285 | 0.0555 | 0.5764 | 0.0204 | 0.0157 | 0.0079 | 0.0520 | 0.0282 | 0.0210 | 0.0001 | 0.0352 | 0.0648 | 0.0472 | 0.1069 |
| KC185405 | *Vertigo pusilla* | 2 | 0.0219 | 0.1344 | 6.1309 | 0.0295 | | 0.0028 | 0.0108 | 0.0208 | 0.0144 | 0.0292 | 0.0268 | 0.0140 | 0.0277 | 0.0242 | 0.0361 | 0.2774 |
| KC185404 | *Pupilla muscorum* | 2 | 0.0240 | 0.1213 | 5.0586 | 0.0404 | | 0.0032 | 0.0254 | 0.0281 | 0.0307 | 0.0116 | 0.0518 | 0.0334 | 0.0354 | 0.0447 | 0.0353 | 0.2856 |
| KC185403 | *Gastrocopta cristata* | 2 | 0.0247 | 0.1131 | 4.5873 | 0.0169 | 0.2923 | 0.0058 | 0.0072 | 0.0151 | 0.0368 | 0.0225 | 0.0312 | 0.0353 | 0.0315 | 0.0478 | 0.0406 | 0.0643 |
| GU475132 | *Platevindex mortoni* | 2 | 0.0161 | 0.0696 | 4.3287 | 0.0137 | | 0.0033 | 0.0087 | 0.0100 | 0.0075 | 0.0082 | 0.0049 | 0.5115 | 0.0093 | 0.0138 | 0.0174 | 0.0038 |
| JN619346 | *Peronia peronii* | 2 | 0.0123 | 0.0549 | 4.4618 | 0.0118 | 0.6669 | 0.0038 | 0.0001 | 0.0103 | 0.0124 | 0.0091 | 0.0061 | 0.0764 | 0.0149 | 0.0042 | 0.0198 | 0.0213 |

| | | | | | | | | | | | | | | | | | | |
|---|---|---|---|---|---|---|---|---|---|---|---|---|---|---|---|---|---|---|
| JN619347 | *Rhopalocaulis grandidieri* | 2 | 0.1031 | 0.2936 | 2.8464 | 0.1129 | 0.0166 | 0.0471 | 0.1030 | 0.1081 | 0.0782 | 0.1278 | 0.1139 | 0.1721 | 0.0771 | 0.1588 | 0.1444 | 0.1910 |
| JN615140 | *Pedipes pedipes* | 2 | 0.0700 | 0.1999 | 2.8554 | 0.0509 | 0.8395 | 0.0178 | 0.0820 | 0.0405 | 0.0511 | 0.0630 | 0.1117 | 0.0847 | 0.0904 | 0.1328 | 0.0817 | 0.1238 |
| JN615139 | *Ovatella vulcani* | 2 | 0.0497 | 0.1252 | 2.5196 | 0.0165 | 0.0001 | 0.0024 | 0.0419 | 0.0309 | 0.0385 | 0.0340 | 0.0882 | 0.0637 | 0.0670 | 0.1662 | 0.0609 | 0.0970 |
| JN606067 | *Myosotella myosotis* | 2 | 0.0866 | 0.2615 | 3.0181 | 0.1228 | 0.0122 | 0.0279 | 0.0477 | 0.0898 | 0.0953 | 0.0472 | 0.0885 | 0.0266 | 0.1220 | 0.1792 | 0.0687 | 0.0065 |
| AP013073 | *Sepia apama* | 3 | 0.0264 | 0.0286 | 1.0854 | | | 0.0024 | 0.0182 | 0.0117 | 0.0166 | | 0.0106 | 0.0129 | 0.0341 | 0.0926 | 0.0234 | 0.0123 |
| AB266516 | *Sepia esculenta* | 3 | 0.0234 | 0.0289 | 1.2360 | 0.0199 | | 0.0027 | 0.0245 | 0.0259 | 0.0168 | 0.0309 | 0.0415 | 0.0068 | 0.0166 | 0.0203 | 0.0236 | 0.0418 |
| AP013074 | *Sepia latimanus* | 3 | 0.0254 | 0.0370 | 1.4577 | 0.0098 | 0.0784 | 0.0039 | 0.0136 | 0.0156 | 0.0175 | 0.0107 | 0.0220 | 0.0403 | 0.0283 | 0.0600 | 0.0195 | 0.0247 |
| KJ162574 | *Sepia lycidas* | 3 | 0.0239 | 0.0215 | 0.8994 | 0.0596 | 0.0558 | 0.0018 | 0.0144 | 0.0241 | 0.0151 | 0.0285 | 0.0473 | 0.0161 | 0.0214 | 0.0600 | 0.0148 | 0.0131 |
| AB240155 | *Sepia officinalis* | 3 | 0.0196 | 0.0214 | 1.0919 | 0.0098 | 0.0151 | 0.0017 | 0.0023 | 0.0200 | 0.0108 | 0.0255 | 0.0142 | 0.0264 | 0.0328 | 0.0064 | 0.0226 | 0.0395 |
| KC632521 | *Sepia pharaonis* | 3 | 0.0198 | 0.0187 | 0.9482 | 0.0128 | 0.0926 | 0.0008 | 0.0172 | 0.0104 | 0.0090 | 0.0234 | 0.0740 | 0.0390 | 0.0159 | | 0.0136 | 0.0053 |
| KF690633 | *Sepia aculeata* | 3 | 0.0241 | 0.0230 | 0.9549 | 0.0212 | 0.0206 | 0.0001 | 0.0105 | 0.0116 | 0.0038 | 0.0078 | 0.0356 | 0.0187 | 0.0167 | 0.0107 | 0.0268 | 0.0126 |
| KF040369 | *Sepiella inermis* | 3 | 0.0185 | 0.0049 | 0.2646 | 0.0115 | 0.0903 | 0.0036 | 0.0053 | 0.3012 | 0.0185 | 0.0240 | 0.0131 | 0.0001 | 0.0122 | 0.0000 | 0.0579 | 0.0345 |
| AB675082 | *Sepiella japonica* | 3 | N*dN < 20 | 0.0006 | 0.0093 | 0.0001 | 0.0000 | 0.0001 | 0.0001 | 0.0001 | 0.0001 | 0.0000 | 0.0001 | 0.0000 | | 0.0000 | 0.0001 | 0.0000 |
| KR912215 | *Sepiella maindroni* | 3 | N*dN < 20 | 0.0007 | 0.0071 | 0.0001 | 0.0000 | 0.0001 | 0.0000 | 0.0001 | 0.0001 | 0.0001 | 0.0000 | 0.0000 | 0.0001 | 0.0000 | 0.3268 | 0.0000 |
| KF647895 | *Idiosepius* sp. | 3 | 0.0074 | 0.0625 | 2.3742 | 0.0092 | 0.2218 | 0.0034 | 0.0003 | 0.0402 | 0.0091 | 0.0134 | 0.0141 | 0.0065 | 0.0146 | 0.0484 | 0.0162 | 0.0135 |
| AP012226 | *Semirossia patagonica* | 3 | 0.0290 | 0.0552 | 1.9028 | 0.0466 | 0.2693 | 0.0079 | | 0.0454 | 0.0177 | 0.0201 | 0.0271 | 0.0621 | 0.0111 | 0.1341 | 0.0288 | 0.0163 |
| GQ225110 | *Loligo opalescens* | 3 | N*dN < 20 | 0.0010 | 0.0000 | | | | | | | | | | | | | |
| AB029616 | *Loligo bleekeri* | 3 | 0.0258 | 0.0256 | 0.9943 | 0.0098 | 0.0096 | 0.0286 | 0.0051 | 0.0427 | 0.0035 | 0.0042 | 0.0615 | 0.0132 | 0.0171 | 0.0058 | 0.0124 | 0.0093 |
| KM878671 | *Sepioteuthis lessoniana* | 3 | 0.0187 | 0.0382 | 2.0472 | 0.0037 | 0.0594 | 0.0020 | 0.0056 | 0.0109 | 0.0013 | 0.0075 | 0.0073 | 0.0139 | 0.0254 | 0.0098 | 0.0173 | 0.0159 |
| KP336703 | *Doryteuthis opalescens* | 3 | N*dN < 20 | 0.0010 | 0.0000 | | | | | | | | | | | | | |
| KT254309 | *Loliolus beka* | 3 | 0.0140 | 0.0051 | 0.3672 | 0.0048 | 0.0205 | 0.0001 | 0.0001 | 0.0181 | 0.0104 | | 0.0242 | 0.0037 | 0.0224 | 0.0001 | 0.0138 | |
| KP265013 | *Loliolus uyii* | 3 | 0.0119 | 0.0059 | 0.4987 | 0.0001 | 0.1403 | 0.0001 | 0.0185 | 0.0096 | 0.0030 | 0.0002 | 0.0280 | 0.0001 | 0.0066 | 0.0277 | 0.0091 | |
| KT362380 | *Uroteuthis chinensis* | 3 | 0.0122 | 0.0087 | 0.7190 | 0.0132 | 0.0344 | 0.0001 | 0.0071 | 0.0140 | 0.0048 | 0.0044 | 0.0129 | 0.0001 | 0.0137 | 0.0200 | 0.0060 | 0.0110 |
| KR051264 | *Uroteuthis duvaucelii* | 3 | 0.0126 | 0.0122 | 0.9679 | 0.0065 | 0.0303 | 0.0008 | 0.0048 | 0.0084 | 0.0027 | 0.0073 | 0.0313 | 0.0090 | 0.0208 | 0.0307 | 0.0156 | 0.0017 |

| | | | | | | | | | | | | | | | | | | |
|---|---|---|---|---|---|---|---|---|---|---|---|---|---|---|---|---|---|---|
| AB675080 | *Uroteuthis edulis* | 3 | 0.0178 | 0.0068 | 0.6951 | 0.0091 | 0.0001 | 0.0001 | 0.0092 | 0.0173 | 0.0017 | 0.0071 | 0.0262 | 0.0113 | 0.0112 | 0.0001 | 0.0114 | 0.0067 |
| KC701763 | *Architeuthis dux* | 3 | 0.0223 | 0.0212 | 0.9529 | 0.0220 | 0.1035 | 0.0008 | 0.0051 | 0.0084 | 0.0101 | 0.0187 | 0.0227 | 0.0120 | 0.0464 | 0.0071 | 0.0183 | 0.0254 |
| AP012225 | *Bathyteuthis abyssicola* | 3 | 0.0296 | 0.0354 | 1.1978 | 0.0275 | 0.0000 | 0.0048 | 0.0249 | 0.0117 | 0.0168 | 0.0204 | 0.0185 | 0.0889 | 0.0412 | 0.0019 | 0.0288 | 0.0104 |
| KJ845633 | *Watasenia scintillans* | 3 | 0.0267 | 0.0605 | 2.2626 | 0.0203 | 0.0528 | 0.0040 | 0.0086 | 0.0107 | 0.0096 | 0.0053 | 0.0294 | 0.0125 | 0.0334 | 0.0415 | 0.0117 | 0.0132 |
| AB715401 | *Ommastrephes bartramii* | 3 | 0.0146 | 0.0171 | 1.1667 | 0.0179 | | 0.0001 | 0.0082 | 0.0001 | 0.0011 | 0.0099 | 0.0181 | 0.0137 | 0.0280 | 0.0096 | 0.0093 | 0.0064 |
| EU068697 | *Dosidicus gigas* | 3 | 0.0139 | 0.0084 | 0.6065 | 0.0001 | 0.0001 | 0.0018 | 0.0123 | 0.0136 | 0.0070 | 0.0063 | 0.0235 | 0.0061 | 0.0272 | 0.0161 | 0.0106 | 0.0077 |
| EU660576 | *Sthenoteuthis oualaniensis* | 3 | 0.0074 | 0.0074 | 0.9989 | 0.0117 | 0.0084 | 0.0001 | 0.0001 | 0.0001 | 0.0007 | 0.0016 | 0.0173 | 0.0237 | 0.0098 | 0.0150 | 0.0070 | 0.0090 |
| AB240153 | *Todarodes pacificus* | 3 | 0.0249 | 0.0120 | 2.2626 | 0.0346 | 0.0635 | 0.0059 | 0.0086 | 0.0344 | 0.0040 | 0.0025 | 0.0092 | 0.0179 | 0.0210 | 0.0070 | 0.0085 | 0.0001 |
| KP336702 | *Illex argentinus* | 3 | 0.0169 | 0.0120 | 0.7055 | 0.1533 | 0.0205 | 0.0130 | 0.0027 | 0.0373 | 0.0016 | 0.0013 | 0.0086 | 0.0258 | 0.0201 | 0.0001 | 0.0098 | 0.0080 |
| KT428877 | *Amphioctopus aegina* | 3 | 0.0360 | 0.0279 | 0.7762 | 0.0215 | 0.0152 | 0.0001 | 0.0094 | 0.0094 | 0.0263 | 0.0225 | 0.0327 | 0.0115 | 0.0443 | 0.0527 | 0.0302 | 0.0167 |
| AB240156 | *Amphioctopus fangsiao* | 3 | 0.0309 | 0.0263 | 0.8500 | 0.0046 | 0.0001 | 0.0019 | 0.0149 | 0.0127 | 0.0140 | 0.0252 | 0.1044 | 0.0116 | 0.0237 | 0.0041 | 0.0184 | 0.0837 |
| KF017606 | *Cistopus chinensis* | 3 | 0.0402 | 0.0228 | 0.5665 | 0.0455 | 0.2468 | 0.0069 | 0.0384 | 0.0172 | 0.0121 | 0.0254 | 0.0774 | 0.0748 | 0.0431 | 0.0637 | 0.0362 | 0.0067 |
| KF017605 | *Cistopus taiwanicus* | 3 | 0.0278 | 0.0183 | 0.6570 | 0.0093 | 0.0718 | 0.0032 | 0.0106 | 0.0193 | 0.0085 | 0.0182 | 0.0972 | 0.0512 | 0.0349 | 0.0082 | 0.0427 | 0.0171 |
| KT581981 | *Octopus bimaculatus* | 3 | 0.0426 | 0.0323 | 0.7576 | 0.0676 | 0.0094 | 0.0302 | 0.0036 | 0.0056 | 0.0179 | 0.0153 | 0.0950 | 0.0181 | 0.0658 | 0.0217 | 0.0426 | |
| KJ789854 | *Octopus conispadiceus* | 3 | 0.0228 | 0.0387 | 1.6976 | 0.0222 | 0.0812 | 0.0042 | 0.0088 | 0.0216 | 0.0226 | 0.0104 | 0.0238 | 0.0025 | 0.0234 | 0.0166 | 0.0266 | 0.0068 |
| HQ638215 | *Octopus minor* | 3 | 0.0238 | 0.0391 | 1.6426 | 0.0239 | 0.0135 | 0.0024 | 0.0204 | 0.0147 | 0.0120 | | 0.0310 | 0.0858 | 0.0286 | 0.0569 | 0.0052 | 0.0010 |
| AB158363 | *Octopus vulgaris* | 3 | 0.0269 | 0.0204 | 0.7577 | 0.0089 | 0.0405 | 0.0039 | 0.0042 | 0.0027 | 0.0164 | 0.0243 | 0.0278 | 0.0122 | 0.0155 | 0.0131 | 0.0402 | 0.0130 |
| AB266515 | *Vampyroteuthis infernalis* | 3 | 0.0122 | 0.0324 | 2.6461 | 0.0480 | 0.3409 | 0.0116 | 0.0142 | 0.0247 | 0.0313 | 0.0280 | 0.0854 | 0.0588 | 0.0354 | 0.0008 | 0.0267 | 0.0017 |
| DQ472026 | *Nautilus macromphalus* | 3 | 0.0441 | 0.0125 | 0.2834 | 0.0345 | 0.1197 | 0.0023 | 0.0219 | 0.0593 | | 0.0287 | 0.0543 | | 0.0558 | 0.0109 | | 0.2397 |
| KP892752 | *Allonautilus scrobiculatus* | 3 | 0.0399 | 0.0082 | 0.2834 | | 0.0000 | 0.0001 | 0.0184 | 0.0001 | 0.0218 | 0.0000 | 0.0000 | 0.0001 | 0.0499 | | 0.0364 | 0.0487 |

Note:

group 1: Poor-migrating group;

group 2: Free-moving group;

group 3: Fast-swimming group.

**Supplementary Table 2.** One nuclear gene (histone H3) in 143 molluscs, and their molecular traits.

| Accession No. | Species | Group | Ka | Ks | Ka/Ks |
|---|---|---|---|---|---|
| HQ009488 | *Crassostrea gigas* | 1 | 0.000016 | 0.160432 | 0.0001 |
| AY654989 | *Arca imbricata* | 1 | 0.000060 | 0.603923 | 0.0001 |
| AY070155 | *Neotrigonia margaritacea* | 1 | 0.000058 | 0.583163 | 0.0001 |
| KP300485 | *Laevichlamys gladysiae* | 1 | 0.000007 | 0.067999 | 0.0001 |
| KP300508 | *Spondylus wrightianus* | 1 | 0.003839 | 0.022688 | 0.1692 |
| KP300507 | *Laevichlamys weberi* | 1 | 0.000010 | 0.097620 | 0.0001 |
| KP300505 | *Complicachlamys wardiana* | 1 | 0.000004 | 0.036130 | 0.0001 |
| HM630385 | *Mizuhopecten yessoensis* | 1 | 0.000020 | 0.195818 | 0.0001 |
| HM622683 | *Azumapecten farreri* | 1 | 0.000000 | 0.000005 | 0.0001 |
| KP300502 | *Swiftopecten swiftii* | 1 | 0.000005 | 0.049822 | 0.0001 |
| HM630481 | *Mimachlamys senatoria* | 1 | 0.003841 | 0.184521 | 0.0208 |
| KP300483 | *Semipallium fulvicostatum* | 1 | 0.000007 | 0.068905 | 0.0001 |
| KP300499 | *Serratovola pallula* | 1 | 0.000008 | 0.083217 | 0.0001 |
| KP300498 | *Dentamussium obliteratum* | 1 | 0.000001 | 0.000003 | 0.2247 |
| KP300497 | *Cryptopecten nux* | 1 | 0.000000 | 0.000005 | 0.0001 |
| KP300495 | *Mirapecten moluccensis* | 1 | 0.000011 | 0.110132 | 0.0001 |
| KP300494 | *Palliolum minutulum* | 1 | 0.000001 | 0.000002 | 0.5075 |
| KP300492 | *Scaeochlamys livida* | 1 | 0.000001 | 0.011224 | 0.0001 |
| KP300482 | *Delectopecten fosterianus* | 1 | 0.003829 | 0.011397 | 0.3359 |
| EU379526 | *Argopecten purpuratus* | 1 | 0.000001 | 0.013230 | 0.0001 |
| KP300489 | *Veprichlamys kiwaensis* | 1 | 0.000000 | 0.000005 | 0.0001 |
| KP300488 | *Notochlamys hexactes* | 1 | 0.000000 | 0.000005 | 0.0001 |
| KR422800 | *Vasticardium enode* | 1 | 0.000002 | 0.000000 | |
| KR422801 | *Vasticardium elongatum* | 1 | 0.000002 | 0.000000 | |
| KR422802 | *Vasticardium flavum* | 1 | 0.000002 | 0.021294 | 0.0001 |
| KR422804 | *Vasticardium insulare* | 1 | 0.003854 | 0.000004 | |
| KR422806 | *Vasticardium pectiniforme* | 1 | 0.000001 | 0.000001 | |
| KR422809 | *Vasticardium vertebratum* | 1 | 0.000001 | 0.000001 | |
| KR422780 | *Microcardium velatum* | 1 | 0.000025 | 0.253124 | 0.0001 |
| KR422781 | *Microfragum festivum* | 1 | 0.000016 | 0.159544 | 0.0001 |
| KR422782 | *Monodacna colorata* | 1 | 0.000015 | 0.150371 | 0.0001 |
| KR422783 | *Papillicardium papillosum* | 1 | 0.000017 | 0.169447 | 0.0001 |
| KR422785 | *Papyridea aff. crockeri* | 1 | 0.007873 | 0.078630 | 0.1001 |
| KR422786 | *Parvicardium exiguum* | 1 | 0.000003 | 0.029345 | 0.0001 |
| KR422787 | *Parvicardium scriptum* | 1 | 0.000002 | 0.017433 | 0.0001 |
| KR422789 | *Trachycardium belcheri* | 1 | 0.000002 | 0.024847 | 0.0001 |
| KR422790 | *Serripes groenlandicus* | 1 | 0.000010 | 0.098707 | 0.0001 |

| | | | | | |
|---|---|---|---|---|---|
| KP300480 | *Talochlamys dichroa* | 1 | 0.000001 | 0.011278 | 0.0001 |
| KR422792 | *Trachycardium egmontianum* | 1 | 0.000004 | 0.036573 | 0.0001 |
| KR422797 | *Vasticardium angulatum* | 1 | 0.000002 | 0.023135 | 0.0001 |
| KR422799 | *Vasticardium assimile* | 1 | 0.000003 | 0.032075 | 0.0001 |
| KR422775 | *Lyrocardium lyratum* | 1 | 0.000008 | 0.076308 | 0.0001 |
| KR422774 | *Laevicardium serratum* | 1 | 0.000002 | 0.023634 | 0.0001 |
| KR422772 | *Laevicardium pictum* | 1 | 0.007539 | 0.055400 | 0.1361 |
| KR422767 | *Keenocardium blandum* | 1 | 0.000005 | 0.052359 | 0.0001 |
| KR422766 | *Fulvia undatopicta* | 1 | 0.000006 | 0.063405 | 0.0001 |
| KR422763 | *Fulvia nienkeae* | 1 | 0.000005 | 0.046056 | 0.0001 |
| KR422761 | *Fulvia mutica* | 1 | 0.000006 | 0.059218 | 0.0001 |
| AY377775 | *Haliotis tuberculata* | 2 | 0.000012 | 0.120310 | 0.0001 |
| AY377778 | *Crepidula fornicata* | 2 | 0.000028 | 0.276938 | 0.0001 |
| EU274516 | *Saulea vitrea* | 2 | 0.000029 | 0.291709 | 0.0001 |
| EU274514 | *Pomacea bridgesi* | 2 | 0.000003 | 0.031949 | 0.0001 |
| EU274513 | *Pila polita* | 2 | 0.000001 | 0.010261 | 0.0001 |
| EU274512 | *Pila conica* | 2 | 0.000000 | 0.000005 | 0.0000 |
| EU274511 | *Marisa cornuarietis* | 2 | 0.000001 | 0.008742 | 0.0001 |
| EU274510 | *Lanistes varicus* | 2 | 0.000013 | 0.129191 | 0.0001 |
| EU274509 | *Lanistes ovum* | 2 | 0.000000 | 0.000004 | 0.0604 |
| EU274507 | *Lanistes nyassanus* | 2 | 0.000000 | 0.000005 | 0.0001 |
| EU274505 | *Afropomus balanoidea* | 2 | 0.000012 | 0.121836 | 0.0001 |
| EU274503 | *Viviparus contectus* | 2 | 0.007604 | 0.165074 | 0.0461 |
| EU274502 | *Bellamya rubicunda* | 2 | 0.000014 | 0.135835 | 0.0001 |
| EU274515 | *Pomacea canaliculata* | 2 | 0.000015 | 0.001789 | 0.0084 |
| KF535893 | *Bellamya lapillorum* | 2 | 0.000001 | 0.010350 | 0.0001 |
| KF535889 | *Bellamya angularis* | 2 | 0.000004 | 0.003730 | 0.0011 |
| KT164405 | *Leptoxis ampla* | 2 | 0.000002 | 0.015886 | 0.0001 |
| JX156678 | *Chilostoma adelozona* | 2 | 0.000001 | 0.012404 | 0.0001 |
| JX156672 | *Causa holosericea* | 2 | 0.000000 | 0.000005 | 0.0000 |
| JX156667 | *Chilostoma subaii* | 2 | 0.000004 | 0.037694 | 0.0001 |
| JX156709 | *Cylindrus obtusus* | 2 | 0.000010 | 0.104941 | 0.0001 |
| JX156657 | *Chilostoma lefeburiana* | 2 | 0.000000 | 0.000005 | 0.0000 |
| JX156652 | *Arianta chamaeleon* | 2 | 0.000003 | 0.025193 | 0.0001 |
| KC660054 | *Dendronotus robustus* | 2 | 0.000015 | 0.153736 | 0.0001 |
| KC660051 | *Dendronotus lacteus* | 2 | 0.000003 | 0.031885 | 0.0001 |
| KM397086 | *Dendronotus niveus* | 2 | 0.000000 | 0.000005 | 0.0000 |
| KM103253 | *Pseudopomatias maasseni* | 2 | 0.000118 | 1.175541 | 0.0001 |
| KP113655 | *Onchidoris muricata* | 2 | 0.000048 | 0.479381 | 0.0001 |
| KP113646 | *Margarites groenlandicus* | 2 | 0.000053 | 0.532188 | 0.0001 |

| | | | | | |
|---|---|---|---|---|---|
| KP113567 | *Flabellina nobilis* | 2 | 0.000063 | 0.630973 | 0.0001 |
| KP113561 | *Limacina helicina* | 2 | 0.003605 | 0.383389 | 0.0094 |
| KP113560 | *Lacuna vincta* | 2 | 0.000033 | 0.332216 | 0.0001 |
| KJ530680 | *Boreotrophon clathratus* | 2 | 0.000027 | 0.273059 | 0.0001 |
| KJ095032 | *Chilostoma ziegleri* | 2 | 0.000001 | 0.014856 | 0.0001 |
| KJ095031 | *Helicigona lapicida lapicida* | 2 | 0.000004 | 0.038173 | 0.0001 |
| KJ095029 | *Cattania inflata* | 2 | 0.000004 | 0.037482 | 0.0001 |
| KJ095025 | *Dinarica serbica* | 2 | 0.000003 | 0.025030 | 0.0001 |
| KJ095024 | *Dinarica pouzolzi* | 2 | 0.000002 | 0.024990 | 0.0001 |
| KJ095017 | *Causa holosericea* | 2 | 0.000000 | 0.000005 | 0.0000 |
| KJ095014 | *Campylaea illyrica* | 2 | 0.000010 | 0.012900 | 0.0008 |
| KJ095012 | *Arianta arbustorum* | 2 | 0.000000 | 0.000005 | 0.0000 |
| JX098189 | *Erjavecia bergeri* | 2 | 0.000007 | 0.074780 | 0.0001 |
| JX098188 | *Macrogastra plicatula* | 2 | 0.000001 | 0.014971 | 0.0001 |
| JX098187 | *Pseudofusulus varians* | 2 | 0.000009 | 0.093201 | 0.0001 |
| JX098186 | *Ruthenica filograna filograna* | 2 | 0.000011 | 0.105227 | 0.0001 |
| KT164569 | *Pleurocera prasinata* | 2 | 0.000006 | 0.063605 | 0.0001 |
| KF535847 | *Bellamya turritus* | 2 | 0.003709 | 0.010482 | 0.3538 |
| KF535846 | *Bellamya dispiralis* | 2 | 0.000002 | 0.000000 | |
| AY557424 | *Sepioteuthis lessoniana* | 3 | 0.000050 | 0.499430 | 0.0001 |
| AY557411 | *Argonauta nodosa* | 3 | 0.000022 | 0.216874 | 0.0001 |
| AY377783 | *Architeuthis dux* | 3 | 0.008804 | 0.145583 | 0.0605 |
| AY557413 | *Grandeledone verrucosa* | 3 | 0.000003 | 0.029492 | 0.0001 |
| AY557414 | *Thaumeledone guntheri* | 3 | 0.000003 | 0.029150 | 0.0001 |
| AY557406 | *Stauroteuthis syrtensis* | 3 | 0.004847 | 0.346436 | 0.0140 |
| AY557407 | *Opisthoteuthis sp.* | 3 | 0.000037 | 0.374274 | 0.0001 |
| AY557408 | *Vampyroteuthis infernalis* | 3 | 0.000027 | 0.265636 | 0.0001 |
| AY557416 | *Heteroteuthis hawaiiensis* | 3 | 0.000000 | 0.000005 | 0.0000 |
| AY557419 | *Stoloteuthis leucoptera* | 3 | 0.000002 | 0.022630 | 0.0001 |
| AY557418 | *Sepiola affinis* | 3 | 0.000008 | 0.083149 | 0.0001 |
| AY557417 | *Rossia palpebrosa* | 3 | 0.000002 | 0.022053 | 0.0001 |
| AY557415 | *Sepia officinalis* | 3 | 0.000010 | 0.096884 | 0.0001 |
| AY557420 | *Spirula spirula* | 3 | 0.000009 | 0.089433 | 0.0001 |
| AY557421 | *Idiosepius pygmaeus* | 3 | 0.000023 | 0.233617 | 0.0001 |
| EU735431 | *Planctoteuthis levimana* | 3 | 0.000002 | 0.023173 | 0.0001 |
| AY557423 | *Loligo pealei* | 3 | 0.000004 | 0.039301 | 0.0001 |
| AY557430 | *Cranchia scabra* | 3 | 0.004543 | 0.164782 | 0.0276 |
| AY557431 | *Leachia atlantica* | 3 | 0.000022 | 0.219934 | 0.0001 |
| AY557432 | *Cycloteuthis syrventi* | 3 | 0.000000 | 0.000005 | 0.0000 |
| AY557433 | *Discoteuthis laciniosa* | 3 | 0.009130 | 0.095494 | 0.0956 |

| | | | | | |
|---|---|---|---|---|---|
| AY557435 | *Enoploteuthis leptura* | 3 | 0.000030 | 0.296088 | 0.0001 |
| AY557436 | *Ornithoteuthis antillarum* | 3 | 0.000027 | 0.269160 | 0.0001 |
| AY557439 | *Gonatus antarcticus* | 3 | 0.018694 | 0.069798 | 0.2678 |
| AY557440 | *Gonatus fabricii* | 3 | 0.000020 | 0.199285 | 0.0001 |
| AY557441 | *Histioteuthis corona* | 3 | 0.000000 | 0.000005 | 0.0000 |
| AY557442 | *Histioteuthis hoylei* | 3 | 0.000003 | 0.032082 | 0.0001 |
| AY557443 | *Histioteuthis reversa* | 3 | 0.000002 | 0.023301 | 0.0001 |
| AY557444 | *Joubiniteuthis portieri* | 3 | 0.000011 | 0.108931 | 0.0001 |
| AY557446 | *Mastigoteuthis agassizii* | 3 | 0.000003 | 0.026960 | 0.0001 |
| AY557447 | *Mastigoteuthis magna* | 3 | 0.000008 | 0.084476 | 0.0001 |
| AY557448 | *Neoteuthis thielei* | 3 | 0.004282 | 0.047872 | 0.0894 |
| AY557449 | *Octopoteuthis sicula* | 3 | 0.004293 | 0.047863 | 0.0897 |
| AY557450 | *Illex coindeti* | 3 | 0.000000 | 0.000005 | 0.0000 |
| AY557451 | *Ommastrephes bartrami* | 3 | 0.004360 | 0.000004 | |
| AY557452 | *Sthenoteuthis oualeniensis* | 3 | 0.000001 | 0.012765 | 0.0001 |
| AY557453 | *Moroteuthis knipovitchi* | 3 | 0.000014 | 0.138847 | 0.0001 |
| AY557454 | *Psychroteuthis sp.* | 3 | 0.004328 | 0.078958 | 0.0548 |
| EU735404 | *Sepiella inermis* | 3 | 0.000010 | 0.096897 | 0.0001 |
| EU735405 | *Lolliguncula diomedeae* | 3 | 0.000005 | 0.054449 | 0.0001 |
| EU735421 | *Gonatopsis sp.* | 3 | 0.000010 | 0.095446 | 0.0001 |
| EU735419 | *Pterygioteuthis giardi* | 3 | 0.000050 | 0.495444 | 0.0001 |
| EU735440 | *Magnapinna sp.* | 3 | 0.000012 | 0.122754 | 0.0001 |
| EU735439 | *Octopoteuthis megaptera* | 3 | 0.000000 | 0.000005 | 0.0000 |
| EU735438 | *Octopoteuthis danae* | 3 | 0.000000 | 0.000005 | 0.0000 |
| EU735423 | *Berryteuthis magister* | 3 | 0.004281 | 0.103198 | 0.0415 |
| EU735426 | *Histioteuthis bonellii* | 3 | 0.000002 | 0.022920 | 0.0001 |

Note:
group 1: Poor-migrating group;
group 2: Free-moving group;
group 3: Fast-swimming group.

**Supplementary Table 3.** The ATP8 gene in 166 molluscs, and their molecular traits.

| Accestion No. | Species | Ka/Ks | Groups | Average Ka/Ks |
|---|---|---|---|---|
| AB809077 | *Fulvia mutica* | 0.61300 | | |
| KF534717 | *Laternula elliptica* | 0.01140 | | |
| KC768038 | *Ostrea lurida* | 0.29180 | | |
| JQ970425 | *Perna viridis* | 0.01300 | | |
| KF214684 | *Mimachlamys senatoria* | 0.38120 | | |
| DQ632742 | *Hiatella arctica* | 0.01650 | | |
| GU071281 | *Meretrix lamarckii* | 0.00010 | | |
| JF969278 | *Paphia undulata* | 0.00010 | Marine bivalve species | 0.1722 |
| JF969276 | *Paphia amabilis* | 0.01620 | | |
| KC832317 | *Meretrix lyrata* | 0.00720 | | |
| EF043342 | *Lucinella divaricata* | 0.10320 | | |
| EF043341 | *Loripes lacteus* | 0.17520 | | |
| KR856227 | *Crassostrea gasar* | 0.27270 | | |
| KR862368 | *Calyptogena magnifica* | 0.08030 | | |
| KP756905 | *Limnoperna fortunei* | 0.20050 | | |
| KM233636 | *Brachidontes exustus* | 0.57350 | | |
| KF296320 | *Solenaia oleivora* | 0.11800 | | |
| AY365193 | *Lampsilis ornata* | 0.02500 | | |
| HM347668 | *Hyriopsis cumingii* | 0.17410 | | |
| KC848654 | *Solenaia carinatus* | 0.17830 | | |
| HM856639 | *Toxolasma parvus* | 0.01350 | | |
| HM856638 | *Lasmigona compressa* | 0.05660 | | |
| HM856635 | *Utterbackia peninsularis* | 0.01070 | | |
| HM856634 | *Margaritifera falcata* | 0.06000 | | |
| HM014130 | *Unio pictorum* | 0.19120 | | |
| FJ809754 | *Pyganodon grandis* | 0.00010 | | |
| FJ809752 | *Venustaconcha ellipsiformis* | 0.11200 | | |
| JQ728447 | *Solemya velum* | 0.56770 | Freshwater bivalve species | 0.0988 |
| JX050180 | *Lamprotula coreana* | 0.39990 | | |
| KM657954 | *Unio douglasiae* | 0.07820 | | |
| KM580067 | *Panopea generosa* | 0.00010 | | |
| KF514426 | *Dahurinaia dahurica* | 0.00010 | | |
| KJ144818 | *Arconaia lanceolata* | 0.00010 | | |
| KJ018924 | *Lamprotula gottschei* | 0.01430 | | |
| KF667529 | *Anodonta lucida* | 0.09790 | | |
| KP187851 | *Anodonta euscaphys* | 0.07350 | | |
| KT247374 | *Potomida littoralis* | 0.00010 | | |
| KT723012 | *Leptodea leptodon* | 0.00010 | | |
| KP273584 | *Cuneopsis pisciculus* | 0.10090 | | |
| KJ472483 | *Haliotis laevigata* | 0.00010 | | |
| KF724723 | *Haliotis discus hannai* | 0.00430 | Gastropodas species | 0.1453 |
| HQ832672 | *Haliotis diversicolor* | 0.04080 | | |

| | | |
|---|---|---|
| FJ605487 | *Haliotis tuberculata* | 0.08560 |
| FJ599667 | *Haliotis tuberculata tuberculata* | 0.00010 |
| KT207826 | *Tegula lividomaculata* | 0.00010 |
| KR297248 | *Angaria neglecta* | 0.03710 |
| KF700096 | *Lunella aff. cinerea* | 0.00170 |
| LC107880 | *Lepetodrilus nux* | 0.07460 |
| KR297250 | *Lepetodrilus schrolli* | 0.00000 |
| KR297249 | *Granata lyrata* | 0.32400 |
| KT207825 | *Diodora graeca* | 0.10670 |
| JN790612 | *Fissurella volcano* | 0.37320 |
| AP013032 | *Chrysomallon squamiferum* | 0.47270 |
| KR297251 | *Phasianella solida* | 0.02260 |
| KF728890 | *Nerita versicolor* | 0.06730 |
| GU810158 | *Nerita melanotragus* | 0.18720 |
| KF728889 | *Nerita tessellata* | 0.00000 |
| KF728888 | *Nerita fulgurans* | 0.00000 |
| KF148053 | *Aplysia kurodai* | 0.00010 |
| AY569552 | *Aplysia californica* | 0.00010 |
| DQ991927 | *Aplysia dactylomela* | 0.08130 |
| AY083457 | *Roboastra europaea* | 0.02330 |
| JN620539 | *Melibe leonina* | 0.00010 |
| JN627205 | *Salinator rhamphidia* | 0.51290 |
| AY345049 | *Siphonaria gigas* | 0.21080 |
| AY345022 | *Siphonaria pectinata* | 0.35780 |
| EU599581 | *Ascobulla fragilis* | 0.00010 |
| AY345048 | *Elysia chlorotica* | 0.51800 |
| JN632509 | *Trimusculus reticulatus* | 0.00340 |
| AB028237 | *Pupa strigosa* | 0.67210 |
| KP716634 | *Naticarius hebraeus* | 0.01430 |
| KM245630 | *Strombus gigas* | 0.00190 |
| HM174254 | *Eualetes tulipa* | 0.00250 |
| HM174253 | *Dendropoma maximum* | 0.09040 |
| HM174252 | *Dendropoma gregarium* | 0.06340 |
| HM174255 | *Thylacodes squamigerus* | 0.26000 |
| KT962044 | *Buccinum pemphigus* | 0.07840 |
| KF897830 | *Babylonia lutosa* | 0.07890 |
| HQ416443 | *Babylonia areolata* | 0.03230 |
| KT382829 | *Volutharpa perryi* | 0.18180 |
| KM603509 | *Varicinassa variciferus* | 0.00010 |
| EU827201 | *Nassarius reticulatus* | 0.00010 |
| EU827195 | *Cancellaria cancellata* | 0.14350 |
| KT199301 | *Conus tribblei* | 0.10440 |
| KR006970 | *Conus tulipa* | 0.01330 |
| DQ862058 | *Conus textile* | 0.11160 |

| | | |
|---|---|---|
| KF887950 | *Conus consors* | 0.27620 |
| EU827198 | *Conus borgesi* | 0.02820 |
| EU827197 | *Fusiturris similis* | 0.13240 |
| DQ284754 | *Lophiotoma cerithiformis* | 0.04770 |
| EU827196 | *Terebra dimidiata* | 0.01290 |
| KM213962 | *Rapana venosa* | 0.00010 |
| JQ446041 | *Concholepas concholepas* | 0.01020 |
| DQ159954 | *Reishia clavigera* | 0.00000 |
| EU827194 | *Bolinus brandaris* | 0.09680 |
| EU827199 | *Cymbium olla* | 0.16340 |
| GU196685 | *Amalda northlandica* | 0.01660 |
| KF736848 | *Semisulcospira libertina* | 0.50800 |
| KC757644 | *Ifremeria nautilei* | 0.07870 |
| DQ238599 | *Lottia digitalis* | 0.27120 |
| JN564796 | *Galba pervia* | 0.72790 |
| KP098541 | *Radix balthica* | 0.00180 |
| KP098540 | *Radix auricularia* | 0.15040 |
| KP279638 | *Radix swinhoei* | 0.00010 |
| JQ390526 | *Physella acuta* | 0.00000 |
| KP279639 | *Planorbarius corneus* | 0.00010 |
| AY380567 | *Biomphalaria glabrata* | 0.00000 |
| EF433576 | *Biomphalaria tenagophila* | 0.00010 |
| KM503121 | *Cipangopaliduna cathayensis* | 0.20960 |
| KM100140 | *Marisa cornuarietis* | 0.39960 |
| KJ739609 | *Pomacea canaliculata* | 0.32760 |
| KR350466 | *Pomacea maculata* | 0.00010 |
| EU079378 | *Oncomelania hupensis robertsoni* | 0.06200 |
| EU001660 | *Oncomelania hupensis hupensis* | 0.00000 |
| FJ997214 | *Oncomelania hupensis* | 0.35220 |
| GQ996415 | *Potamopyrgus estuarinus* | 0.00000 |
| GQ996430 | *Potamopyrgus antipodarum* | 0.00010 |
| KM365085 | *Cerion incanum* | 0.45970 |
| KJ744205 | *Achatina fulica* | 0.01950 |
| KM083123 | *Mastigeulota kiangsinensis* | 0.18090 |
| KT192071 | *Aegista aubryana* | 0.00600 |
| JQ417194 | *Helix aspersa* | 0.11950 |
| JN107636 | *Cylindrus obtusus* | 0.21000 |
| KT821554 | *Naesiotus nux* | 0.74140 |
| NC_001761 | *Albinaria caerulea* | 0.57640 |
| KC185403 | *Gastrocopta cristata* | 0.29230 |
| JN619346 | *Peronia peronii* | 0.66690 |
| JN619347 | *Rhopalocaulis grandidieri* | 0.01660 |
| JN615140 | *Pedipes pedipes* | 0.83950 |
| JN615139 | *Ovatella vulcani* | 0.00010 |

| | | | | |
|---|---|---|---|---|
| JN606067 | *Myosotella myosotis* | 0.01220 | | |
| AP013074 | *Sepia latimanus* | 0.07840 | | |
| KJ162574 | *Sepia lycidas* | 0.05580 | | |
| AB240155 | *Sepia officinalis* | 0.01510 | | |
| KC632521 | *Sepia pharaonis* | 0.09260 | | |
| KF690633 | *Sepia aculeata* | 0.02060 | | |
| KF040369 | *Sepiella inermis* | 0.09030 | | |
| AB675082 | *Sepiella japonica* | 0.00000 | | |
| KR912215 | *Sepiella maindroni* | 0.00000 | | |
| KF647895 | *Idiosepius* sp. | 0.22180 | | |
| AP012226 | *Semirossia patagonica* | 0.26930 | | |
| AB029616 | *Loligo bleekeri* | 0.00960 | | |
| KM878671 | *Sepioteuthis lessoniana* | 0.05940 | | |
| KT254309 | *Loliolus beka* | 0.02050 | | |
| KP265013 | *Loliolus uyii* | 0.14030 | | |
| KT362380 | *Uroteuthis chinensis* | 0.03440 | | |
| KR051264 | *Uroteuthis duvaucelii* | 0.03030 | | |
| AB675080 | *Uroteuthis edulis* | 0.00010 | | |
| KC701763 | *Architeuthis dux* | 0.10350 | Cephalopods species | 0.0665 |
| AP012225 | *Bathyteuthis abyssicola* | 0.00000 | | |
| KJ845633 | *Watasenia scintillans* | 0.05280 | | |
| EU068697 | *Dosidicus gigas* | 0.00010 | | |
| EU660576 | *Sthenoteuthis oualaniensis* | 0.00840 | | |
| AB240153 | *Todarodes pacificus* | 0.06350 | | |
| KP336702 | *Illex argentinus* | 0.02050 | | |
| KT428877 | *Amphioctopus aegina* | 0.01520 | | |
| AB240156 | *Amphioctopus fangsiao* | 0.00010 | | |
| KF017606 | *Cistopus chinensis* | 0.24680 | | |
| KF017605 | *Cistopus taiwanicus* | 0.07180 | | |
| KT581981 | *Octopus bimaculatus* | 0.00940 | | |
| KJ789854 | *Octopus conispadiceus* | 0.08120 | | |
| HQ638215 | *Octopus minor* | 0.01350 | | |
| AB158363 | *Octopus vulgaris* | 0.04050 | | |
| AB266515 | *Vampyroteuthis infernalis* | 0.34090 | | |
| DQ472026 | *Nautilus macromphalus* | 0.11970 | | |
| KP892752 | *Allonautilus scrobiculatus* | 0.00000 | | |

Marine bivalve species vs. Freshwater bivalve species, P=0.188

Marine bivalve species vs. Gastropodas species, P=0.628

Marine bivalve species vs. Cephalopods species, P=0.062

**Supplementary Figure 1** The ML tree based on (A) *ATP6*, (B) CoxI, (C) CoxII and (D) Cytb gene in molluscs

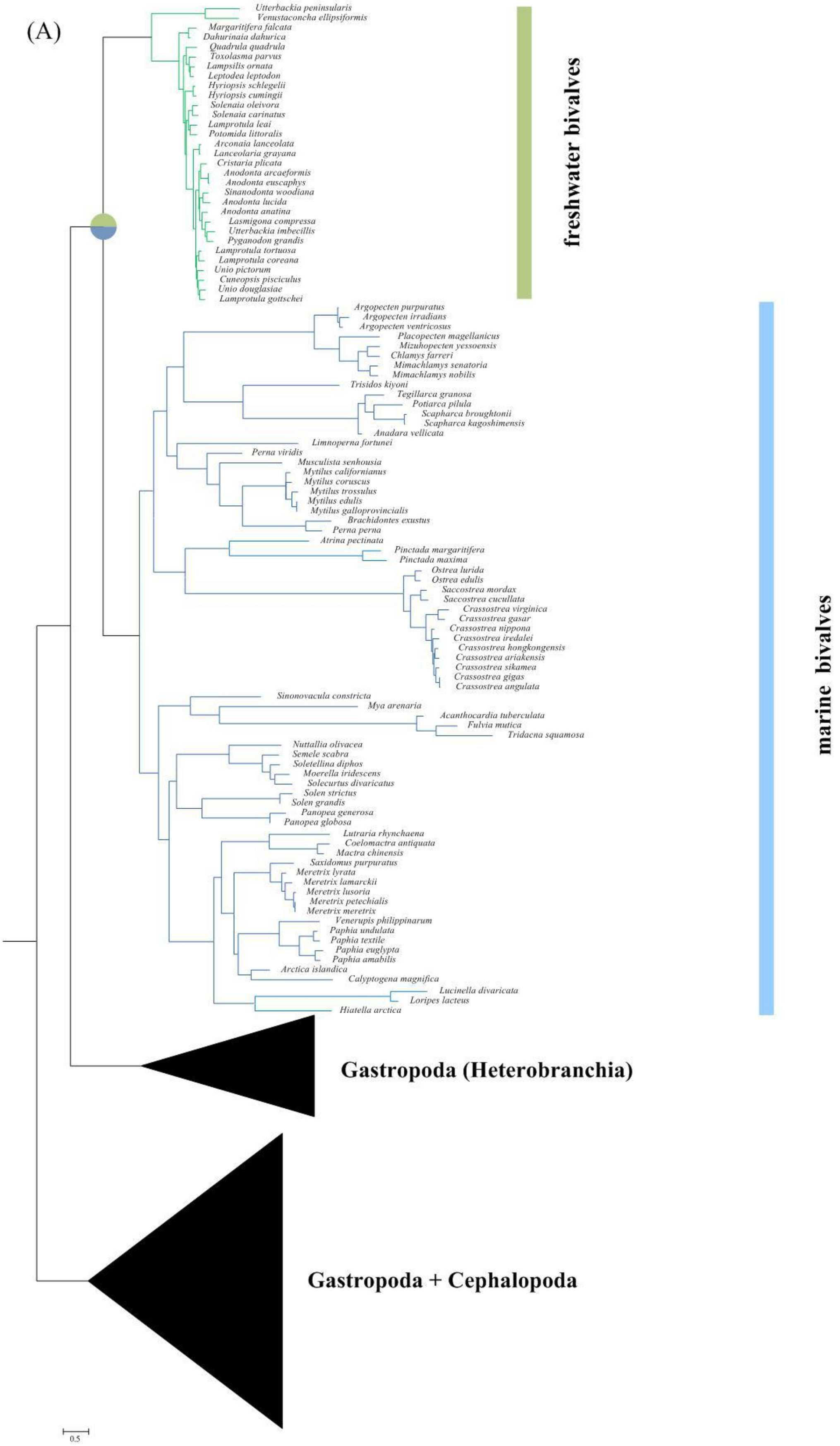

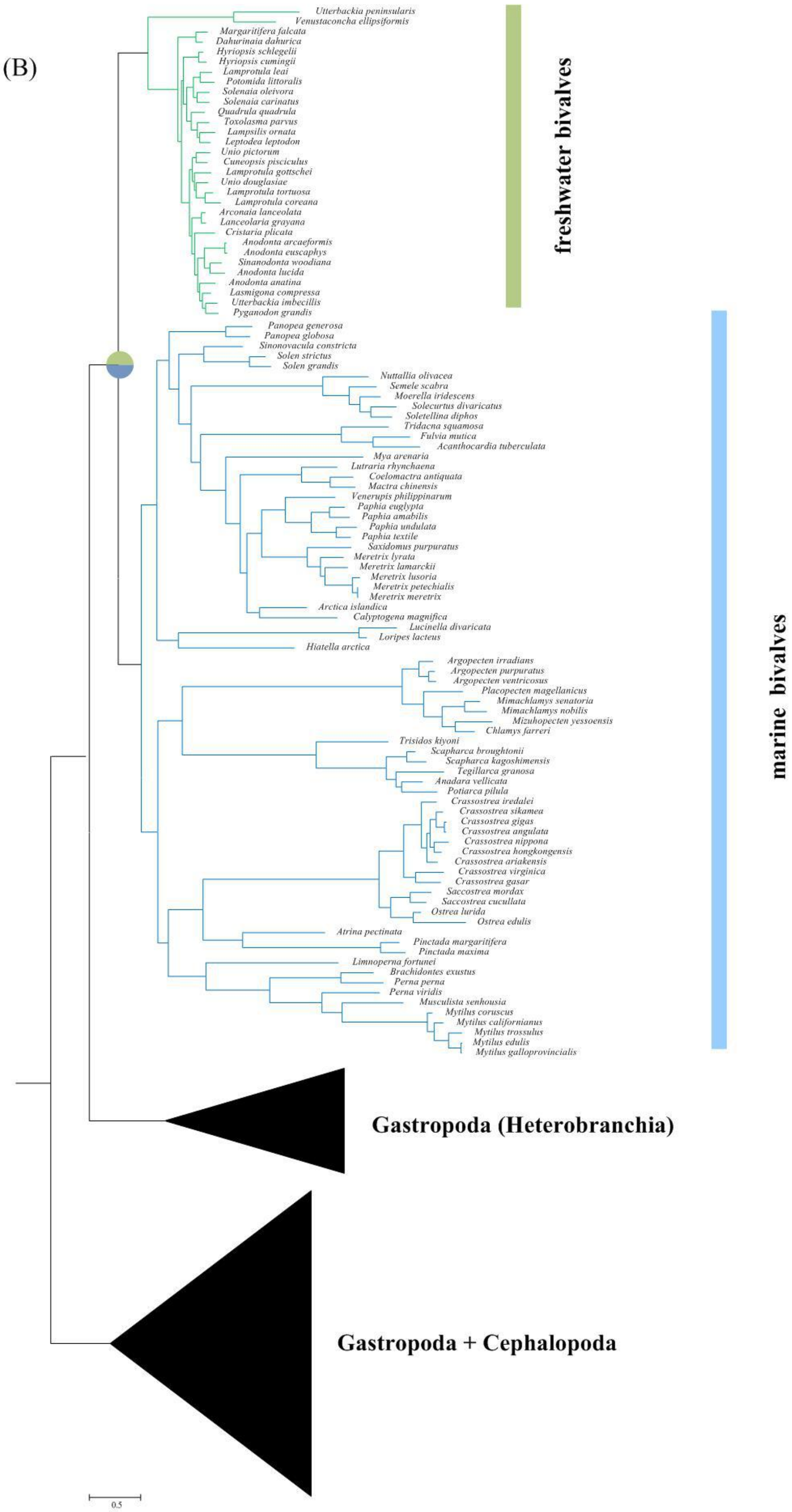

(B)
Utterbackia peninsularis
Venustaconcha ellipsiformis
Margaritifera falcata
Dahurinaia dahurica
Hyriopsis schlegelii
Hyriopsis cumingii
Lamprotula leai
Potomida littoralis
Solenaia oleivora
Solenaia carinatus
Quadrula quadrula
Toxolasma parvus
Lampsilis ornata
Leptodea leptodon
Unio pictorum
Cuneopsis pisciculus
Lamprotula gottschei
Unio douglasiae
Lamprotula tortuosa
Lamprotula coreana
Arconaia lanceolata
Lanceolaria grayana
Cristaria plicata
Anodonta arcaeformis
Anodonta euscaphys
Sinanodonta woodiana
Anodonta lucida
Anodonta anatina
Lasmigona compressa
Utterbackia imbecillis
Pyganodon grandis
freshwater bivalves
Panopea generosa
Panopea globosa
Sinonovacula constricta
Solen strictus
Solen grandis
Nuttallia olivacea
Semele scabra
Moerella iridescens
Solecurtus divaricatus
Soletellina diphos
Tridacna squamosa
Fulvia mutica
Acanthocardia tuberculata
Mya arenaria
Lutraria rhynchaena
Coelomactra antiquata
Mactra chinensis
Venerupis philippinarum
Paphia euglypta
Paphia amabilis
Paphia undulata
Paphia textile
Saxidomus purpuratus
Meretrix lyrata
Meretrix lamarckii
Meretrix lusoria
Meretrix petechialis
Meretrix meretrix
Arctica islandica
Calyptogena magnifica
Lucinella divaricata
Loripes lacteus
Hiatella arctica
Argopecten irradians
Argopecten purpuratus
Argopecten ventricosus
Placopecten magellanicus
Mimachlamys senatoria
Mimachlamys nobilis
Mizuhopecten yessoensis
Chlamys farreri
Trisidos kiyoni
Scapharca broughtonii
Scapharca kagoshimensis
Tegillarca granosa
Anadara vellicata
Potiarca pilula
Crassostrea iredalei
Crassostrea sikamea
Crassostrea gigas
Crassostrea angulata
Crassostrea nippona
Crassostrea hongkongensis
Crassostrea ariakensis
Crassostrea virginica
Crassostrea gasar
Saccostrea mordax
Saccostrea cucullata
Ostrea lurida
Ostrea edulis
Atrina pectinata
Pinctada margaritifera
Pinctada maxima
Limnoperna fortunei
Brachidontes exustus
Perna perna
Perna viridis
Musculista senhousia
Mytilus coruscus
Mytilus californianus
Mytilus trossulus
Mytilus edulis
Mytilus galloprovincialis
marine bivalves
Gastropoda (Heterobranchia)
Gastropoda + Cephalopoda
0.5

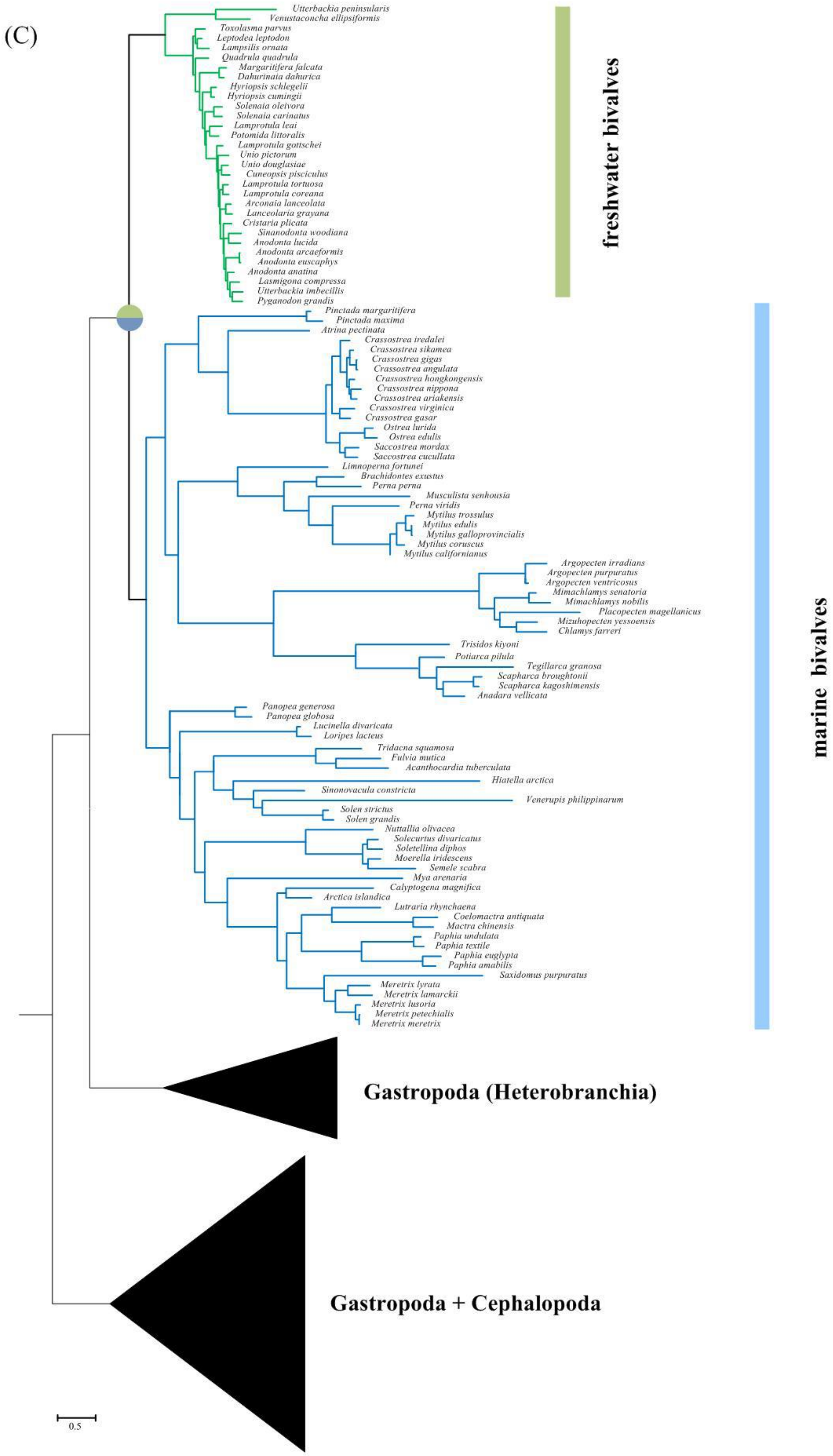
(C)
Utterbackia peninsularis
Venustaconcha ellipsiformis
Toxolasma parvus
Leptodea leptodon
Lampsilis ornata
Quadrula quadrula
Margaritifera falcata
Dahurinaia dahurica
Hyriopsis schlegelii
Hyriopsis cumingii
Solenaia oleivora
Solenaia carinatus
Lamprotula leai
Potomida littoralis
Lamprotula gottschei
Unio pictorum
Unio douglasiae
Cuneopsis pisciculus
Lamprotula tortuosa
Lamprotula coreana
Arconaia lanceolata
Lanceolaria grayana
Cristaria plicata
Sinanodonta woodiana
Anodonta lucida
Anodonta arcaeformis
Anodonta euscaphys
Anodonta anatina
Lasmigona compressa
Utterbackia imbecillis
Pyganodon grandis
freshwater bivalves
Pinctada margaritifera
Pinctada maxima
Atrina pectinata
Crassostrea iredalei
Crassostrea sikamea
Crassostrea gigas
Crassostrea angulata
Crassostrea hongkongensis
Crassostrea nippona
Crassostrea ariakensis
Crassostrea virginica
Crassostrea gasar
Ostrea lurida
Ostrea edulis
Saccostrea mordax
Saccostrea cucullata
Limnoperna fortunei
Brachidontes exustus
Perna perna
Musculista senhousia
Perna viridis
Mytilus trossulus
Mytilus edulis
Mytilus galloprovincialis
Mytilus coruscus
Mytilus californianus
Argopecten irradians
Argopecten purpuratus
Argopecten ventricosus
Mimachlamys senatoria
Mimachlamys nobilis
Placopecten magellanicus
Mizuhopecten yessoensis
Chlamys farreri
Trisidos kiyoni
Potiarca pilula
Tegillarca granosa
Scapharca broughtonii
Scapharca kagoshimensis
Anadara vellicata
Panopea generosa
Panopea globosa
Lucinella divaricata
Loripes lacteus
Tridacna squamosa
Fulvia mutica
Acanthocardia tuberculata
Hiatella arctica
Sinonovacula constricta
Venerupis philippinarum
Solen strictus
Solen grandis
Nuttallia olivacea
Solecurtus divaricatus
Soletellina diphos
Moerella iridescens
Semele scabra
Mya arenaria
Calyptogena magnifica
Arctica islandica
Lutraria rhynchaena
Coelomactra antiquata
Mactra chinensis
Paphia undulata
Paphia textile
Paphia euglypta
Paphia amabilis
Saxidomus purpuratus
Meretrix lyrata
Meretrix lamarckii
Meretrix lusoria
Meretrix petechialis
Meretrix meretrix
marine bivalves
Gastropoda (Heterobranchia)
Gastropoda + Cephalopoda
0.5

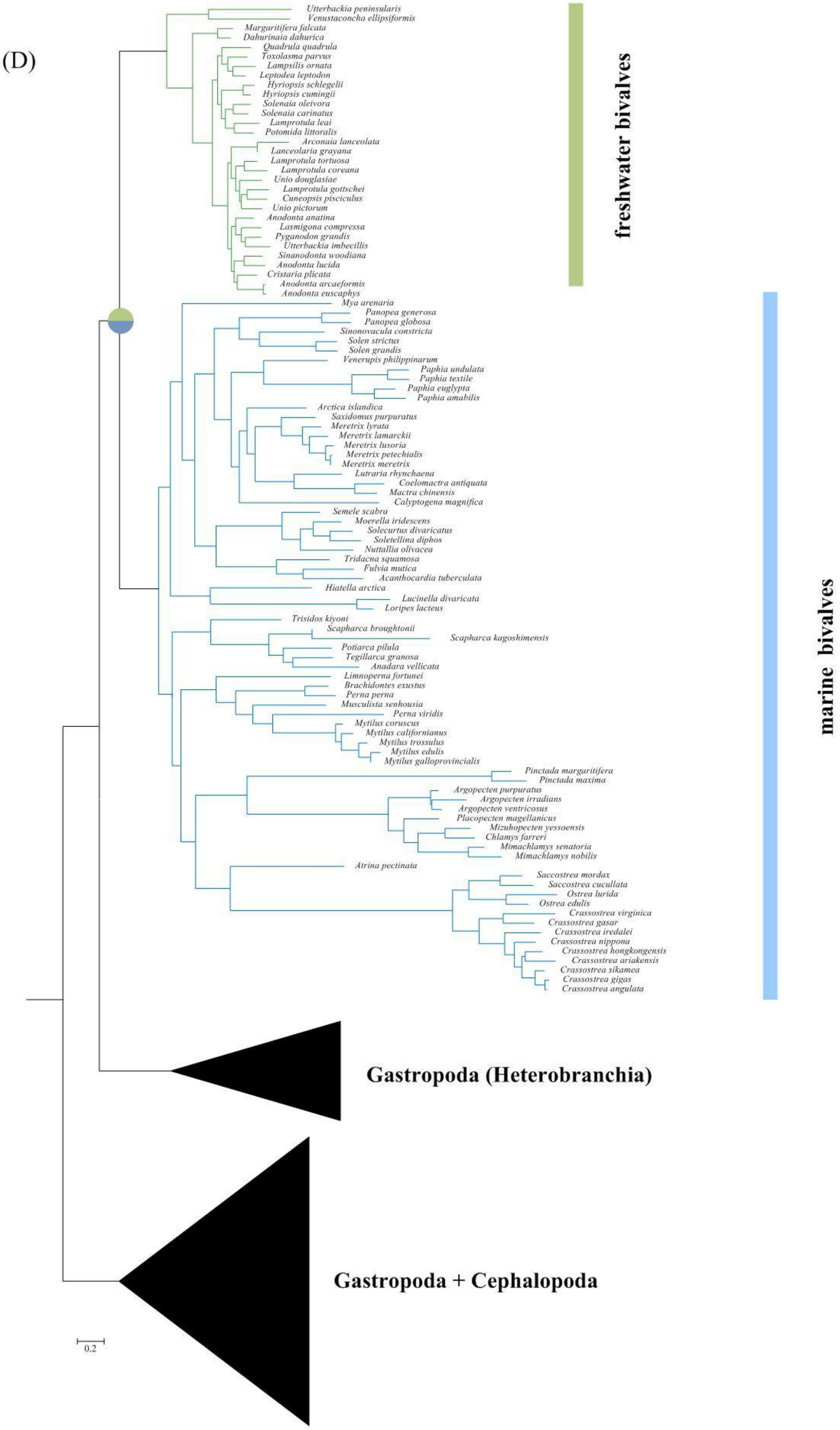
(D)
Utterbackia peninsularis
Venustaconcha ellipsiformis
Margaritifera falcata
Dahurinaia dahurica
Quadrula quadrula
Toxolasma parvus
Lampsilis ornata
Leptodea leptodon
Hyriopsis schlegelii
Hyriopsis cumingii
Solenaia oleivora
Solenaia carinatus
Lamprotula leai
Potomida littoralis
Arconaia lanceolata
Lanceolaria grayana
Lamprotula tortuosa
Lamprotula coreana
Unio douglasiae
Lamprotula gottschei
Cuneopsis pisciculus
Unio pictorum
Anodonta anatina
Lasmigona compressa
Pyganodon grandis
Utterbackia imbecillis
Sinanodonta woodiana
Anodonta lucida
Cristaria plicata
Anodonta arcaeformis
Anodonta euscaphys
freshwater bivalves
Mya arenaria
Panopea generosa
Panopea globosa
Sinonovacula constricta
Solen strictus
Solen grandis
Venerupis philippinarum
Paphia undulata
Paphia textile
Paphia euglypta
Paphia amabilis
Arctica islandica
Saxidomus purpuratus
Meretrix lyrata
Meretrix lamarckii
Meretrix lusoria
Meretrix petechialis
Meretrix meretrix
Lutraria rhynchaena
Coelomactra antiquata
Mactra chinensis
Calyptogena magnifica
Semele scabra
Moerella iridescens
Solecurtus divaricatus
Soletellina diphos
Nuttallia olivacea
Tridacna squamosa
Fulvia mutica
Acanthocardia tuberculata
Hiatella arctica
Lucinella divaricata
Loripes lacteus
Trisidos kiyoni
Scapharca broughtonii
Scapharca kagoshimensis
Potiarca pilula
Tegillarca granosa
Anadara vellicata
Limnoperna fortunei
Brachidontes exustus
Perna perna
Musculista senhousia
Perna viridis
Mytilus coruscus
Mytilus californianus
Mytilus trossulus
Mytilus edulis
Mytilus galloprovincialis
Pinctada margaritifera
Pinctada maxima
Argopecten purpuratus
Argopecten irradians
Argopecten ventricosus
Placopecten magellanicus
Mizuhopecten yessoensis
Chlamys farreri
Mimachlamys senatoria
Mimachlamys nobilis
Atrina pectinata
Saccostrea mordax
Saccostrea cucullata
Ostrea lurida
Ostrea edulis
Crassostrea virginica
Crassostrea gasar
Crassostrea iredalei
Crassostrea nippona
Crassostrea hongkongensis
Crassostrea ariakensis
Crassostrea sikamea
Crassostrea gigas
Crassostrea angulata
marine bivalves
Gastropoda (Heterobranchia)
Gastropoda + Cephalopoda
0.2
